# Management of cardiovascular disease by cerium oxide nanoparticles via alleviating oxidative stress and adipokine abnormalities

**DOI:** 10.1038/s41598-025-85794-6

**Published:** 2025-02-17

**Authors:** Samir A. E. Bashandy, Marawan A. Elbaset, Fatma A. A. Ibrahim, Sahar S. Abdelrahman, Sherif A. Abdelmottaleb Moussa, Ahmed M. A. El-Seidy

**Affiliations:** 1 Pharmacology Department, Medical Research and Clinical Studies Institute, National Research Centre, El-bohouth St., P.O. 12622 Dokki, Cairo Egypt; 2 Department of Biochemistry, Biotechnology Research Institute, National Research Centre, El-bohouth St., P.O. 12622 Dokki, Cairo Egypt; 3 Pathology Department, Faculty of Veterinary Medicine, Cairo University, Giza, Egypt; 4 Inorganic Chemistry Department, Advanced Materials Technology & Mineral Resources Research Institute, National Research Centre, El-bohouth St., P.O. 12622 Dokki, Cairo Egypt

**Keywords:** Cerium oxide nanoparticles, Obesity, Aorta, Cardiac muscle, Adipokines, Oxidative stress, Insulin resistance, Bioinorganic chemistry, Biochemistry, Biological techniques, Inorganic chemistry

## Abstract

The current study aimed to evaluate the role of cerium oxide nanoparticles (C-1), a potent antioxidant, in the medication of cardiovascular disease in obese animal model. C-1 was prepared using a modified sonication sol-gel method. Thirty-two adult male rats were equally divided into 4 groups (n=8/each). The first (control) and second (obese) groups are not treated while the obese rats in the third and fourth groups were given 15 and 30 mg/kg C-1(IP), respectively, for 8 weeks. Parameters of insulin resistance, adipocyte hormones, inflammatory markers, lipid profile, cardiac enzymes and cardiac iron content (C-Fe) were estimated. Moreover, histological study and immunohistochemical stain for inducible nitric oxide synthase (INOS) for cardiac and aortic tissues were performed. The XRD patterns of C-1 showed narrow symmetric diffraction peaks. The particle diameters were calculated from the TEM histogram (21.09 nm) and the Debye-Scherrer Method (20.74 nm) which were very similar. Using the most intense peak ($28.47^{\circ }$), structural parameters were calculated including nano-crystallite size, Micro-strain, Lorentz factor, Thomson polarization parameter, and Lorentz polarization parameter. BET was used to calculate The total surface area (S$_{T}$), and specific surface area (S$_{BET}$). The XPS survey spectrum of C-1 showed peaks for C-1s, O-1s and Ce-3d. The treatment of obese rats with C-1 led to a significant decrease in body weight, C-Fe , plasma leptin, tumor necrosis factor-alpha (TNF$\alpha$), interleukin-6 (IL6), C-reactive protein (CRP), resistin, cholesterol, triglycerides, low-density lipoprotein (LDL), Troponin, Creatinine Kinase-MB (CK-MB), lactate dehydrogenase (LDH), and malondialdehyde (MDA) in cardiac tissue or in plasma. Also, C-1 lowered plasma monocyte chemoattractant protein-1 (MCP-1), Epithelial Neutrophil-Activating Peptide (ENA-78), and insulin and glucose levels in obese rats. Furthermore, C-1 alleviated the increase of cardiac iNOS. Moreover, C-1 mitigated pathological changes of cardiac muscle and aorta observed in obese rats. On the other hand, C-1 enhanced adiponectin, cardiac glutathione (GSH) and superoxide dismutase (SOD) in obese rats. The effect of C-1 is dose-dependent ( 30 mg/kg of C-1 is more evident than 15 mg/kg). The modified synthesis method may lead to a smaller particle size than that reported in our previously reported work. The XRD patterns of C-1 indicate its cubic structure with space group F m -3 m (225) which was matched by code id 4343161 from COD. The Raman spectrum of C-1 indicates the absence of rearrangement oxygen atoms, the presence of oxygen in its fluorite lattice positions, and the oxygen vacancies in C-1 and the Ce vibration model (F_2g_). The presence of ten peaks in the high-resolution Ce-3d XP spectrum indicates the existence of both Ce^3+^ and Ce^4+^. C-1 showed therapeutic efficacy in atherosclerosis and cardiac muscle abnormalities associated with obese rats, probably because of their antioxidant and anti-inflammatory properties, which lead to lowering oxidative stress.

## Introduction

Obesity is a multifactorial disease related to many complications as dyslipidemia, type 2 diabetes, insulin resistance,hypertension, and cardiovascular disease(CVDs). Notably, abdominal obesity, gauged through metrics like waist circumference and visceral adiposity, stands out as a key indicator of heightened susceptibility to CVDs. The onset of obesity globally has resulted in widespread impacts on cardiovascular health, necessitating the need for better prevention and assistance. Increased levels of cholesterol, high blood pressure and coronary artery disease are more prone among people who are overweight and obese^1,2^. There are two kinds of pathways that have roles in insulin resistance development. One is the inflammatory one, while the other is not. Resistance to insulin hampers the metabolism of lipids and carbohydrates in the body. This will cause an increase in fats and make some processes unbalanced. The triggering of insulin resistance eventually leads to an inflamed environment by increasing specific cytokines such as leptins, TNF$\alpha$ and IL6 . The many harmful impacts of insulin resistance are highlighted through this complex series on the cellular and molecular machinery important for controlling metabolism and inflammation^3–5^. The resistance of the cells to insulin supply leads to the clustering of platelet cells and raises chemical levels that promote cell coagulation^6^. Adiponectin and leptin are two important factors that greatly impact cardiovascular health. It’s interesting to note how the data points establish a strong correlation between these two levels and the occurrence of coronary artery disease. Syndromes of this sort are bonded with decreased levels of adiponectin and an increased level of leptin. Adipokines can also be used as markers to identify and treat coronary artery diseases among patients due to the effects of adipose tissue on cardiovascular health. This highlights the importance of these pathways within this system^7,8^. Diseases that are surely associated with obesity, such as CVDs, might occur within the body due to unbalanced levels of radicals and reactive oxygen species by the antioxidizing tool^9^. A high-fat diet leads to reactive oxygen species production by oxidative stress. Atherosclerosis is caused due to lipid peroxidation and accumulates over time^10^. Research has demonstrated that consuming a diet rich in fat leads to an upregulation of nitric oxide synthase (iNOS) or the synthesis of nitric oxide (NO) in many circumstances, particularly in the bloodstream^11^, hepatic cells^12^, and cardiac tissue^11,13^. It is well known that NO generated by iNOS plays a crucial role in the pathophysiology of metabolic dysfunction that is caused by obesity^14^. Experimentally, it has been proven that NO produced by iNOS plays a role in the process of endothelial dysfunction, which has been demonstrated via many different experiments^15^.

There is increasing attention for research on cerium, which is a rare-earth element that is a member of the lanthanide family, because cerium may decrease inflammation in both in vitro and in vivo settings and because it has antioxidant properties^16,17^. Keeping a healthy balance between oxidation and anti-oxidation is thought to be important for the functioning of biological systems. Endocytosis was found to be the process by which cerium oxide nanoparticles (CeO$_{2}$NPs) were taken in and spread out in endothelial cells. This kept the cells safe from oxidative damage. This was found through earlier research. CeO$_{2}$NPs are thought to counter the hydrogen peroxide-induced oxidative stress and apoptosis, making them much less severe^18^. CeO$_{2}$NPs protect against the development of cardiovascular diseases linked inflammation and oxidative stress. It’s possible that CeO$_{2}$NPs’ positive effects are linked to their natural ability to make antioxidants. These results show that CeO$_{2}$ nanoparticles might be useful as a treatment for heart problems and keep the heart functioning well^19^. It is commonly accepted that the dual oxidation state of CeO$_{2}$NPs is the most well-known mechanism underpinning their function^20^. The scientific community is very interested in cerium and its oxides. This includes physics, chemistry, biology, and material sciences, among others. The unique properties that set cerium and its oxides apart from other elements come from the fact that they have empty spaces in their valence shells, especially in the 4f and 5d orbitals. The presence of these vacancies is affected by the chemical environment. This is one of the reasons why cerium compounds are complex and have multiple properties^21^. In contrast, there is a striking lack of information about CeO$_{2}$NPs being helpful for heart disease. This lack of knowledge is even more severe in overweight animals. The research now wants to check how well C-1 medicine works in treating the heart disease of overweight rats.

Therefore, the target of the present study is the appreciation of the therapeutic role of current synthesis CeO$_{2}$NPs (C-1) for combating cardiovascular disease in obese rats via the evaluation of oxidative stress parameters, insulin resistance, inflammatory markers, adipocyte hormones, and chemoattractant protein. We also assess iNOS in cardiac and aortic tissues. We believe that this comprehensive understanding of the complete picture of C-1 could be helpful for this type of setup that may lead to cardiovascular abnormalities.

## Materials and methods

### Material

$\hbox {Ce}(\hbox {SO}_{4})_{2}\cdot \hbox {4H}_{2}\hbox {O}$, KOH and PEG (polyethylene glycol (8000 LR)) were purchased from Sigma Aldrich (USA). All used chemicals were either HPLC analytical grade or high analytical grade.

### Synthesis of C-1

C-1 was synthesized using the sonication-sol-gel method^22,23^. 150 mL DD (double distilled water) containing 15 g cerium(IV) sulfate tetrahydrate $(\hbox {Ce}(\hbox {SO}_{4})_{2}\cdot \hbox {4H}_{2}\hbox {O}$, 37.10 mmol) was added to 100 mL DD containing 2.5 g potassium hydroxide pellets (KOH, 44.56 mmol) and then added to PEG solution (240 mL DD, 1 g of PEG) under sonication for 30 min. The mixture was put under stirring (1000 rpm) at $80 ^{\circ }\hbox {C}$. The resulting solution was completely dried before being crushed into granules. The resulting powders were then crushed after being calcined at $650^{\circ }$C for four hours in an air environment in a furnace. The resulting powder was washed several times with DD and dried (@ $100^{\circ }$C) in an air environment in a furnace.

### Experimental techniques

The structural, crystallite size of the sample was investigated using X-ray diffraction. The XRD patterns were obtained from X’pert PRO diffractometer with a Cu-radiation ($\lambda$ = 1.542Å) at 45 K.V. and 35mA over the range of $2\theta =2^{\circ }- 60^{\circ }$ and the average size of the crystallites was calculated by Debye-Scherrer equation. HR-TEM was carried out using the TEM model JEOL 2100 LB_6_ transmission electron microscope at the National Research Center, Cairo, Egypt. XPS was collected on K-ALPHA (Thermo Fisher Scientific, USA) with monochromatic X-ray Al K-alpha radiation -10 to 1350 e.v spot size 400 micro m at pressure 10^-9^ mbar with full spectrum pass energy 200 e.v and at narrow spectrum 50 e.v. ImageJ was used on TEM to obtain histogram data. Sonication condition: direct immersion, ultrasonic vibracell, 20 kHz, 50 % of 550 wt., temperature below $80 ^{\circ }$C. The methods for measuring surface area are based on gas absorption according to Brunauer-Emmet-Teller (BET) method and non-local density functional theory (NL-DFT) for cylindrical pores was applied to evaluate the pore size distribution (PSD). $\hbox {N}_{2}$ sorption analysis was carried out to obtain the BET surface area (S$_{BET}$). N$_{2}$ physisorption experiments were carried out using the BELSORP MAX, Japan, surface area, and pore size analyzer.

### Experimental protocol

Thirty-two adult male Wistar rats (aged 10 weeks and weighing between 140 and 165 g) were chosen from the Animal House of the National Research Center in Egypt. The rats were accommodated in a stable environment (room temperature of $25^{\circ }$, 12-h light/dark cycle), and given free access of food and water. The animals were left for one week for adaptation. The animal experiments were executed in conformity with instructions for the Care and Use of Laboratory Animals in the National Research Centre and Medical Research Ethics Committee (MREC, Ethics number: 20195).

The experimental design included eight rats designated as the control group. The remaining rats were subjected to a high-fat diet along with water containing 25 % sucrose for a duration of 16 weeks to induce obesity. The composition of the high-fat diet comprised carbohydrates (42.3 %), protein (17 %), fat (22.50 %), fiber (3.2 %), minerals (5 %), and moisture (10 %). Rats in the control group were fed a standard chow pellet without any dietary manipulation. The rats were fed ad libitum and each of the 4 rats were housed in a cage. The body weight of each rat was measured once a week. At the beginning of the treatment of rats, we provided each cage with same quantity of diet (100 g). This experimental setup aimed to establish an obese rat model for subsequent investigations into the effects of obesity and potential therapeutic interventions. The rats were separated into 4 groups, each contains 8 rats: -

- Group 1: The normal rats as a control.
- Group 2: The obese group administered IP in the same volume of vehicle (water).
- Group 3: Obese rats injected with 15 mg/kg C-1 IP injection for 56 days.
- Group 4: Obese rats injected with 30 mg/kg C-1 IP injection for 56 days.

C-1 had a size of around 21 nm. C-1 dosages used were in accordance with Morbidi et al. (2018)’s experimental study^24^. C-1 was suspended in deionized water and dispersed with the aid of a sonicator. The Animal Care and Use rules from the National Research Center in Egypt were followed, along with any other steps that were suggested, when working with the animals in the study. Approved By the National Research Center’s Medical Research Ethics Committee No.19218, animal handling was carried out according to recommendations and under Animal Care and Use of National Research Centre regulations in Egypt and in accordance with the guidelines of the American Veterinary Medical Association (AVMA). Anesthesia was given to all animals that were going to have surgery, and great care was taken to make sure they were in as little pain or suffering as possible. Ethical concerns, following the rules, and using anesthesia showed that the researchers cared about the welfare and humane care of the animals used in the studies. All experiments on living animals were performed under the roles and regulations of the Animal Research: Reporting of In Vivo Experiments (ARRIVE) and the American Veterinary Medical Association (AVMA) Guidelines for the Euthanasia of Animals (2020)

#### Anthropometric measures

Rats’ Body Mass Index (BMI) may be computed using the following formula, which takes into account their height and weight:

1 $$\begin{aligned} BMI = \frac{body \hspace{3pt} weight \hspace{3pt} (g)}{Squared\hspace{3pt}height \hspace{3pt} (cm^{2})}. \end{aligned}$$

The BMI values and waist circumference were measured as part of the rats’ physical evaluation. This method gives us a way to measure the relationship between height and body weight in the rats we are studying. This helps figure out what the rats’ body composition is like and if there are any links to the way the experiment was set up, like a high-fat diet or other changes.

#### Samples

All groups of rats had a 12-h fast at the end of the treatment period. Xylazine (20 mg/kg IP) and Ketamine (50 mg/kg IP) were used in animal anesthesia. Heparinized tubes were used to collect blood samples from a tail vein, and Eppendorf tubes were used to retain the separated plasma at -80° for further biochemical analysis. The animals were immediately killed by cervical dislocation, and the aorta and heart were removed after blood collection. To produce a homogenate, a particular weighted section of the heart was homogenized with ice-cooled saline (0.9 percent NaCl). Using a cooling centrifuge from “Laborzentrifugen, Sigma, Germany”, the homogenate was centrifuged for 10 minutes at $5^{\circ }\hbox {C}$ at 3000 rpm. After that, the supernatant was used for a number of analyses. A portion of the heart and aorta were simultaneously and quickly fixed in 10 % neutral buffered formalin. After the tissues were fixed, they were made ready for light microscopy by cutting paraffin slices into 5 $\mu$m thick pieces. Hematoxylin and eosin (H&E) were used to check the histological features . Some heart and aorta slices were also subjected to an immunohistochemistry study.

#### Determination of iron in tissues

The method described by Imeryuz et al.^25^ was used to measure the iron levels in heart and fat tissues . 60 % perchloric acid and then 60 % nitric acid were used to treat one gram of tissue for thirty minutes together. After that, the samples were spun at 3000 g for 12 min^25^. Using a graphite furnace atomic absorption spectrophotometer, the non-heme iron content of the supernatant was measured after it had been diluted with deionized water. It was measured in mg of non-heme iron per gram of tissue mass, which gave the results. Comparing and measuring the amounts of iron in heart and fat tissues using this method is a reliable way to find out what effects iron levels have in these specific biological settings.

#### Plasma and cardiac oxidative stress parameters

The amounts of MDA , SOD, and GSH were assessed in plasma and heart samples using Biodiagnostic kits from Cairo, Egypt, and colorimetric tests. A colorimetric method was used to find MDA, which is a sign of lipid breakdown. In the test, MDA mixed with a certain substance to make a chromophoric product. The strength of this product could be measured using spectrophotometry. A colorimetric method was used to measure SOD, an enzyme that helps protect cells from free radicals. Stopping an event linked to superoxide radicals could be part of the process. The drop in color intensity would show how active SOD is. Colorimetric research was also used to check the GSH, which is a very important antioxidant for cells. The test involves GSH combining with a certain chemical to make a colored molecule, with the amount of GSH affecting how bright the color is.

#### Plasma inflammatory markers

ELISA was used to find out how many of several biomarkers were present. These included TNF$\alpha$ , IL6 , CRP , Resistin, ENA78 (epithelial neutrophil-activating protein 78), and MCP-1 (monocyte chemoattractant protein-1). Sunlong Biotech Co., Kit, in Hangzhou, China, sold the ELISA kits that were used for these measurements.

Proteins can be found and measured in ELISA using special antibodies. Antibodies and detecting chemicals are added after the target protein is stuck to a stable surface, like a microplate. After the color changes, which shows how much of the target protein is in the sample, the measurement can be very accurate and sensitive.

#### Plasma biomarkers for myocardial function

A kit from Hangzhou, China-based Sunlong Biotech Co. was used to test the troponin using the immunoassay method. When it comes to immunoassays, antibodies are often used to find and measure certain proteins in a sample. Troponin shows how healthy the heart is. We used chemicals from the German company Centronic-gmbh Wartenberg, Germany, to measure the kinetic activity of LDH and CK-MB enzymes, which are used to indicate the health of heart tissues.

#### The adipocyte hormones and lipid profile

The immunoassay approach was used to measure the plasma levels of adiponectin and leptin (ELISA, Sunlong Biotech Co. Kit, Hangzhou, China). The Salucea Company, located in Haansberg, Netherlands, produced the colorimetric kits used to measure cholesterol, triglycerides, HDL, and LDL levels. The equation: TG/HDL-C, was used to compute the atherogenic index.

Higher values of this index, a computed measure that may give insight into the risk of atherosclerosis development, indicate an increased risk. For these crucial metabolic and cardiovascular indicators, complete use of immunoassay and colorimetric techniques using commercial kits from respectable vendors guarantees standardized and trustworthy readings.

#### Insulin resistance parameters

Colorimetric measurement of blood glucose was conducted using the Salucea Company Haansberg, Netherlands, kit instructions. As directed by Sunlong Biotech Co. Kit, an immunoassay (ELISA) was used to measure plasma insulin (Hangzhou, China). The following formula was used to determine the insulin resistance index: -

2 $$\begin{aligned} (HM-IR) = \frac{fasting \hspace{2pt} glucose \hspace{2pt} (mg/dl) \hspace{2pt}x \hspace{5pt} fasting \hspace{2pt} insulin \hspace{2pt} (mIU/ml)}{405}. \end{aligned}$$

Higher values in this index indicate a greater resistance to the effects of insulin, and it serves as an estimate of insulin resistance.

#### Histopathological studies

After being excised, the tissue specimens from the abdominal and cardiac aortas were carefully cleaned by normal saline to remove any blood and immediately placed in buffered neutral formalin. The specimens were fixated for twenty-four hours, and then they were processed normally to produce paraffin blocks. These paraffin blocks were then carefully sectioned into slices that were about 4–5 $\mu$m thick. After that, several staining methods were applied to the sections to improve characterization and visibility. Also, Orcein and Gomori’s Trichrome were used for staining, following the method that Bancroft and Gamble came up with. These staining methods shed light on the shape and make-up of cells, connective tissue, and other parts of the tissue^26^. This detailed histological method lets us look closely at the heart and abdominal aorta tissues. This lets us look into changes in structure, cellular shape, and any abnormal changes that might be important to the study goals.

#### Immunohistochemical studies

This study used the avidin-biotin-peroxidase method (DAB, Sigma-Aldrich, St. Louis, USA) to find iNOS expression in heart samples from all the groups. A diluted iNOS monoclonal antibody from Dako Corp in California, USA, was mixed with paraffin slices. This was done along with the avidin-biotin-peroxidase procedure’s suggested tools (Vactastain ABC peroxidase kit, Vector Laboratories, New Jersey, USA). Colored chromagen 3,3-diaminobenzidine tetrahydrochloride (DAB, Sigma-Aldrich, St. Louis, USA) was used to see the marker expression. We used image analysis software (Image J, 1.46a, NIH, USA, Maryland, USA) to measure the optical density of the positive brown color in 5 tiny fields. A score was given for the level of leptin expression in periaortic fat in white adipocytes with only one eye, brown adipocytes with many eye cells, and differentiated adipocytes: leptin negative (0), mildly positive (I), positive (II), and highly positive (III) (III). The current study used the avidin-biotin-peroxidase method to find iNOS expression in heart samples without using immunohistochemistry. The chromagen 3,3-diaminobenzidine tetrahydrochloride (DAB) and other chemicals needed for this method were bought from Sigma Chemical Co. The antibody used was a monoclonal one against iNOS from Dako Corp. in California, USA. It was diluted 1:200. The staining process involved putting wax slices in a container with the iNOS monoclonal antibody and the recommended avidin-biotin peroxidase kit from Vector Laboratories in New Jersey, USA. The chromagen DAB was then used to see how much of the iNOS marker was present. A computer program called Image J, 1.46a, from the NIH in Maryland, USA, was used to measure the optical density of the positive brown color in five tiny areas. The immunohistochemical approach, along with quantitative analysis, allows for the assessment of iNOS expression in cardiac tissues.

#### Aortic morphometric study

The thickness of the tunica intima, media, and adventitia in the tissue samples was quantified using image analysis software, specifically Image J (version 1.46a, NIH, Maryland, USA). Image J is a widely used software for digital image processing and analysis. The use of image analysis software enhances objectivity and precision in measuring tissue dimensions, contributing to the reliability and reproducibility of the results obtained from the histological analysis of the vascular structures.

#### Statistical analysis

The variability of the results is represented as the mean ± standard error of the mean (SEM). The standard deviation of the data was evaluated using the Brown-Forsythe Test, and the normality was assessed with the Shapiro-Wilk test. The data meeting these criteria were assessed using one-way ANOVA and subsequently ranked with Tukey-Kramer post hoc multiple comparison. If the data did not meet the aforementioned criteria, their median value was displayed and was analyzed using the Kruskal-Wallis test and Dunn’s multiple comparison.

## Results

### C-1

**Figure 1:**
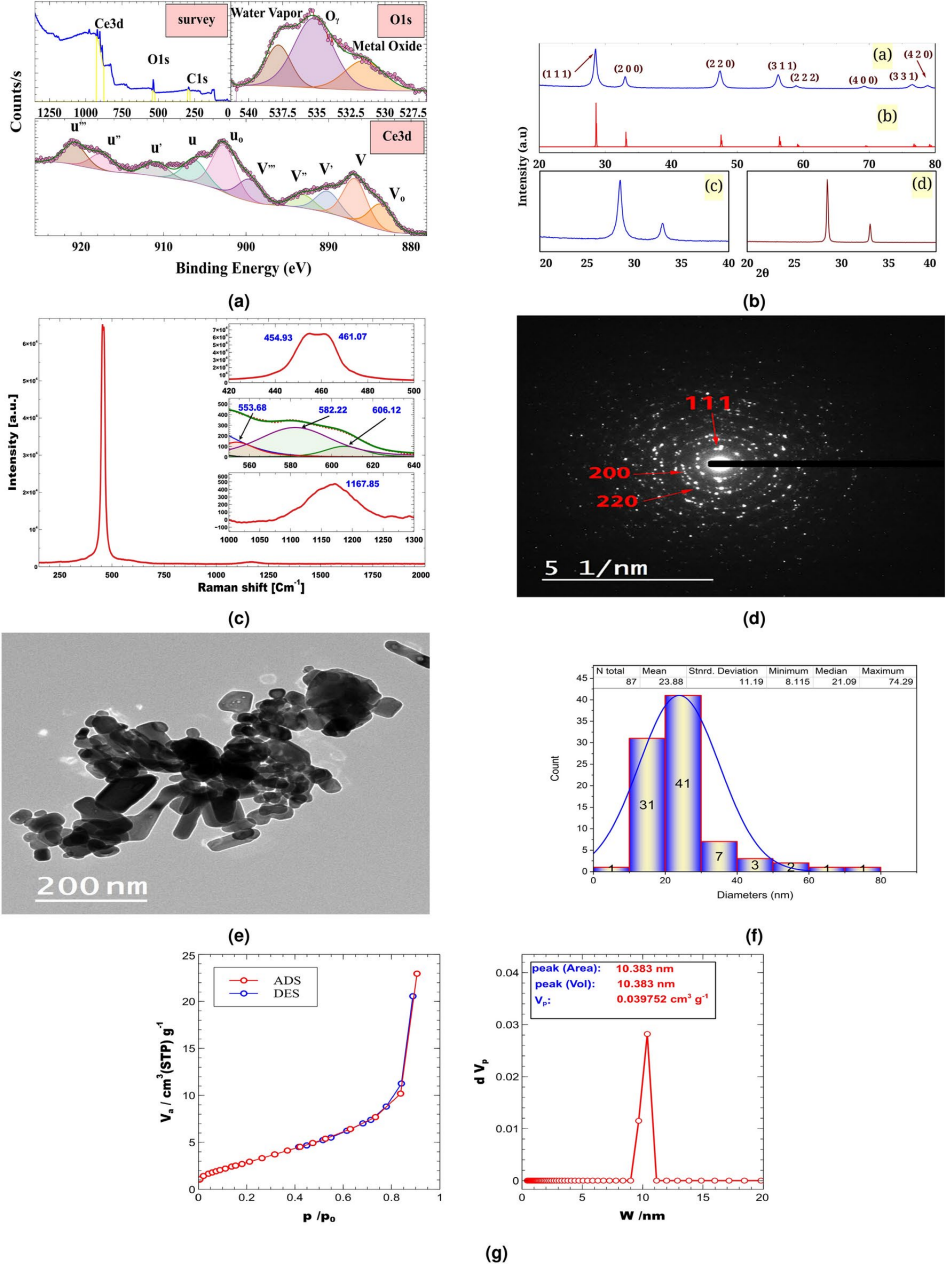
**a**: XPS spectra of C-1, **b**: (**a**) XRD diffractogram of C-1, (**b**) COD:1562989, (**c**) Expantion (20–40) of XRD diffractogram of C-1, (**d**) Expantion (20–40) of XRD diffractogram of (A-1)^23^, **c**: Raman spectrum of C-1, d: HRTEM image of C-1, **e**: TEM image of C-1, **f**: Particle diameters distributions, **g**: N$_{2}$ sorption isotherms and corresponding pore size distributions of C-1.

The XRD patterns of C-1 (Fig. 1b) showed slightly broad symmetric diffraction peaks at $28.52^{\circ }, 32.97^{\circ }, 47.40^{\circ }, 56.19^{\circ }, 58.94^{\circ }, 69.17^{\circ }, 76.52^{\circ }$, and $78.86^{\circ }$. The cell parameters are a = b = c = 5.42 Å and $\alpha$ = $\beta$ = $\gamma = 90^{\circ }$, respectively. The particle diameters distributions were calculated from the TEM histogram data, Fig. 1f. The average particle diameters are about 21.09 nm while that calculated using the Debye-Scherrer Method was 20.74 nm. Using the most intense peak (28.52^o^), structural parameters were calculated including Micro-strain (V = 6.85 x 10^-3^$\epsilon$), Lorentz factor (Lf= 4.25), Thomson polarization parameter (TPp= 0.89), and Lorentz polarization parameter (LPp= 30.13). The XPS survey spectrum of C-1 showed multiple peaks at 902.45, 533.99, and 290.62 eV. The high-resolution (HR) O-1s XP spectrum shows three peaks at 530.85, 534.52, and 537.11 eV while HR Ce-3d XP spectrum shows ten peaks at 883.37, 886.58, 890.06, 893.24, 899.12, 902.28, 905.73, 910.7, 916.58, and 920.26 eV. The HRTEM image, Fig. 1d shows clear (1 1 1), (2 0 0), and (2 2 0) planes. BET isotherm (Fig. 1g) of C-1 was used to calculate S$_{BET}$ (145.78 m$^{2}$), while the pore size distribution analysis (pdn) (Fig. 1g) indicated a mean pore diameter (Mpd) of 10.38 nm. The Raman spectrum (Fig. 1c) of C-1 showed doublet (at 454.93 and 461.07 cm^−1^) , three peaks in 553.68–606.12 cm^−1^ region and a peak at 1167.85 cm^−1^.

### Effect of C-1 on anthropometric measures and visceral adipose tissue weight

**Figure 2:**
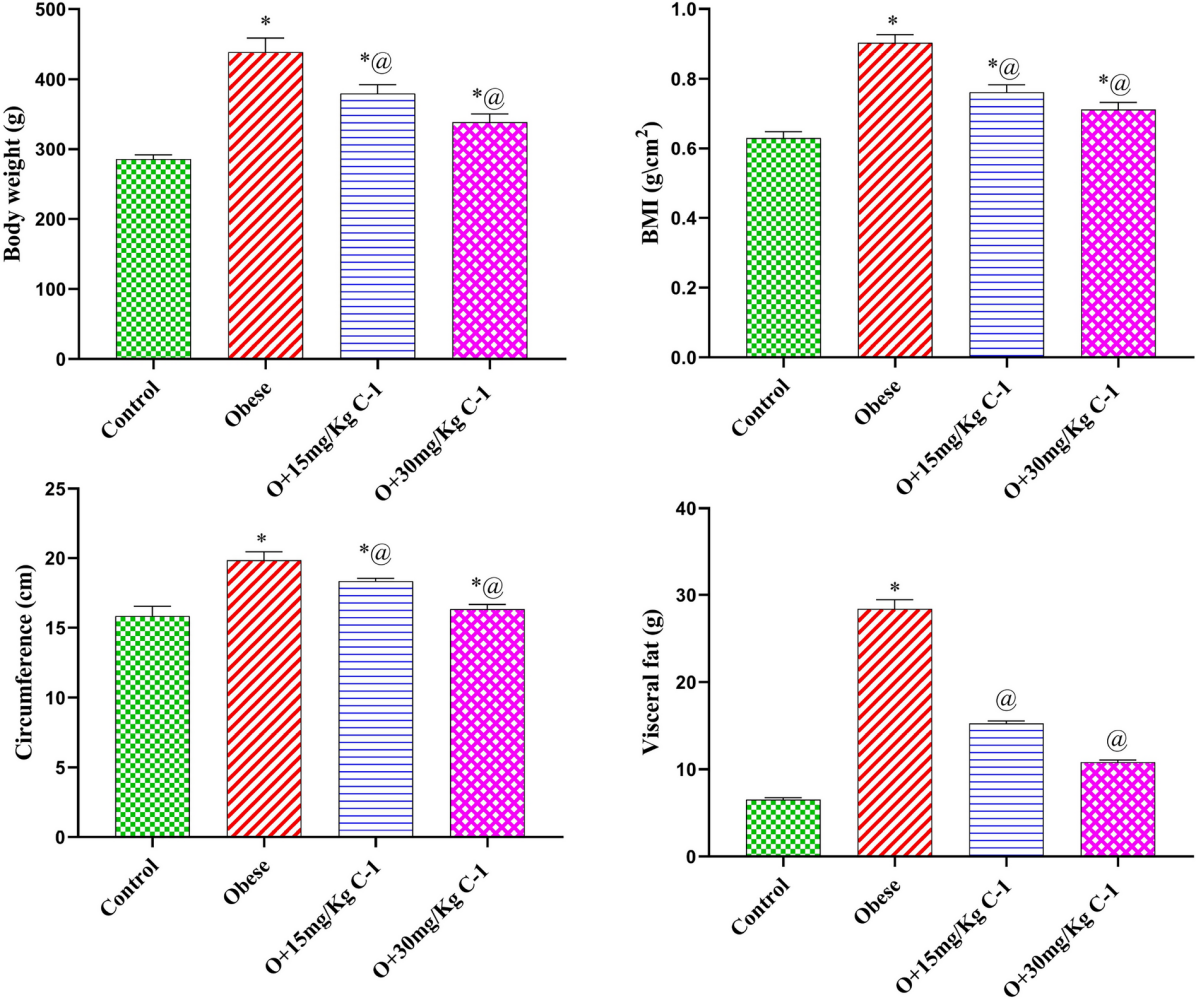
Effect of C-1 on anthropometric measures and visceral adipose tissue weight of obese rats. Each bar represents the mean of 8 animals ± se. Statistical analysis was performed using one-way ANOVA followed by Tukey-Kramer multiple comparisons test. ($\star$ vs control group(group 1), @ vs obese group(group 2) ) at $p<$ 0.05.

**Figure 3:**
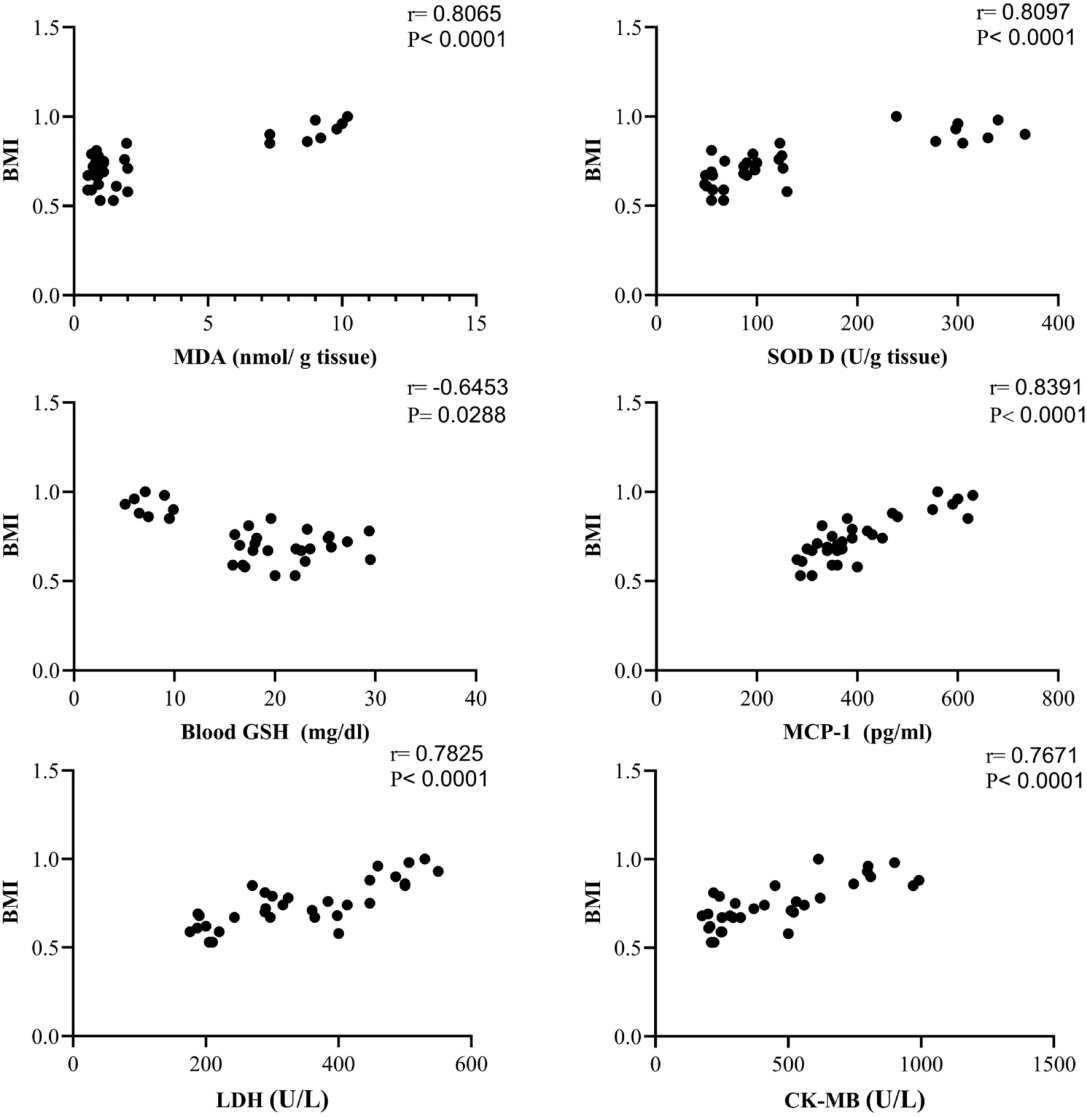
Correlation between BMI and some oxidative parameters and cardiac biomarkers.

As shown in Fig. 2 group 2 displayed a significant increase in body weight, body mass index (BMI), waist circumference (WI), and visceral adipose tissue (VAA) by 53, 43, 25 and 334 % compared to group 1. On the other hand, rats treated with C-1 (15 or 30 mg/kg) showed a diminution in body weight, BMI, WI, and VAA by 13, 16, 8, and 46 % for group 3 and 23, 21, 18, and 61 % for group 4 compared to group 2. Regarding the correlation analysis Fig. 3 there was a positive correlation between BMI and MDA, MCP-1, LDH, and CK-MB. On the otherhand, there was a negative correlation between BMI and GSH. A significant difference was noted between group 1 and treated rats (group 3 and 4) in the values of BMI, body weight, and circumference but the values of visceral fat weight showed no significant difference.

### Effect of C-1 on plasma lipid profile and adipocyte hormones

**Figure 4:**
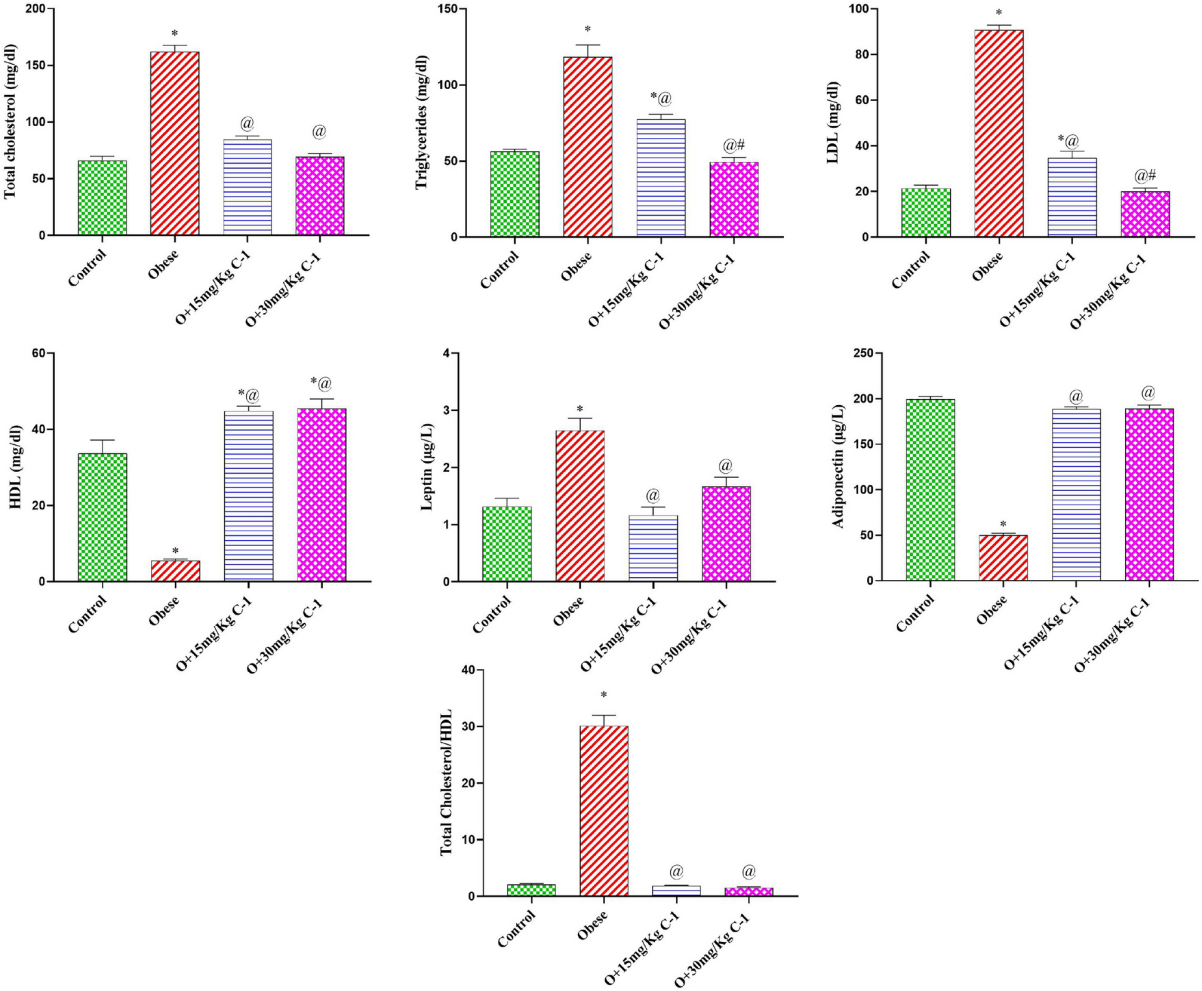
Effect of C-1 on plasma lipid profile and adipocyte hormones of obese rats. Each bar represents the mean of 8 animals ± se. Statistical analysis was performed using one-way ANOVA followed by Tukey-Kramer multiple comparisons test. ($\star$ vs control group(group 1), @ vs obese group(group 2) and # vs Nano cerium oxide (15 mg/kg, group 3)) at $p<0.05$.

Group 2 experienced a rise in total cholesterol, triglycerides, LDL, and leptin by 145, 111, 325, and 101 % compared to group 1 (Fig. 4). On the contrary, the administration of C-1 hindered the rise of total cholesterol, triglycerides, LDL, and leptin by 48, 35, 62 and 37 % for group 3, and by 57, 58, 78 and 56 % for group 4 compared to group 2. In a similar manner, group 2 suffered from low levels of HDL and adiponectin by 83 and 75 % compared to group 1. However, the C-1 increased the HDL and adiponectin levels by 8.1 and 3.7-fold for group 3 and 8.2 and 4-fold for group 4 compared to group 2. Also, C-1 treatment in group 3 and 4 mitigates the increase in atherogenic index (TG/HDL). The levels of total cholesterol, adiponectin, leptin, and atherogenic index (total cholesterol/HDL) in group 3 and 4 did not change significantly relative to group 1 values.

### Effect of C-1 on the iron content of Cardiac and adipose tissue of obese rats

**Figure 5:**
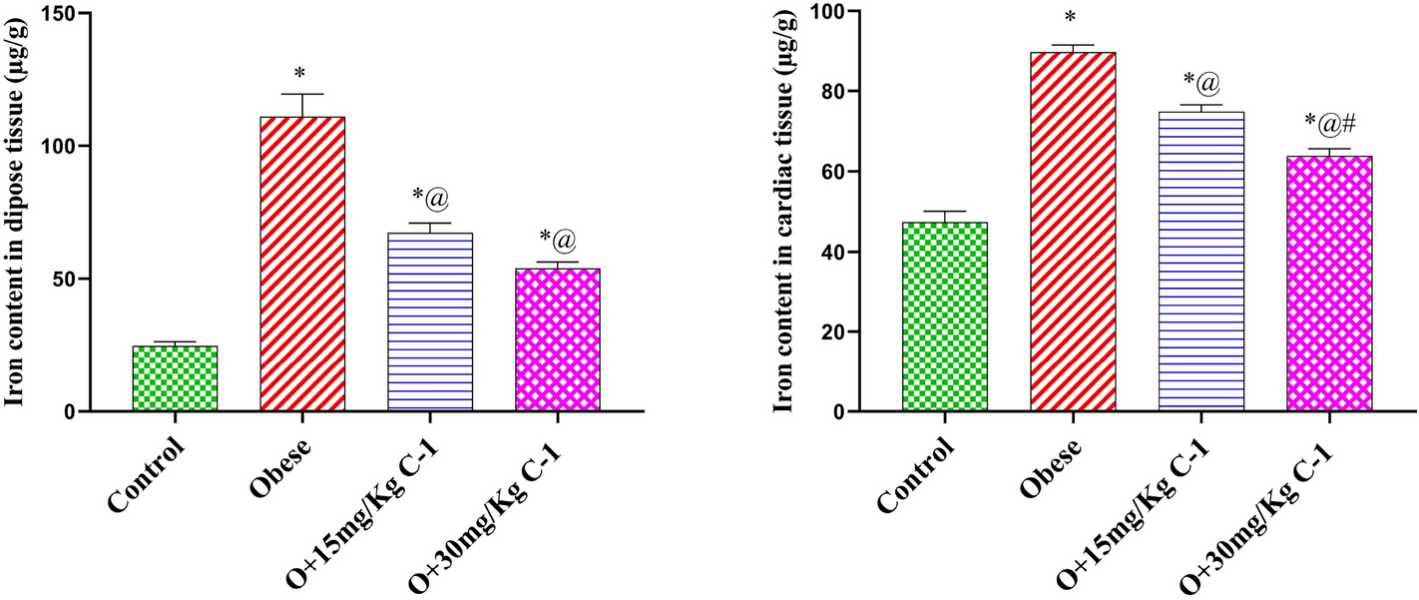
Effect of C-1 on the iron content of cardiac and adipose tissue of obese rats.Each bar represents the mean of 8 animals ± se. Statistical analysis was performed using one-way ANOVA followed by Tukey-Kramer multiple comparisons test. ($\star$ vs control group(group 1), @ vs obese group(group 2) and # vs Nano cerium oxide (15 mg/kg, group 3)) at $p<0.05$.

Group 2 showed high iron contents of adipose and cardiac tissue by 4.5 and 1.9-fold compared to group 1 (Fig. 5). On the other hand, C-1 reduced the iron contents of adipose and cardiac tissue by 39 and 17 % for group 3 and by 51 and 29 % for group 4 compared to the obese rats. The values of Iron content in groups 3 and 4 are higher than that of group 1 but lawer than that the value found for group 2.

### Effect of C-1 on the oxidative stress of plasma and cardiac tissue of obese rats

**Figure 6:**
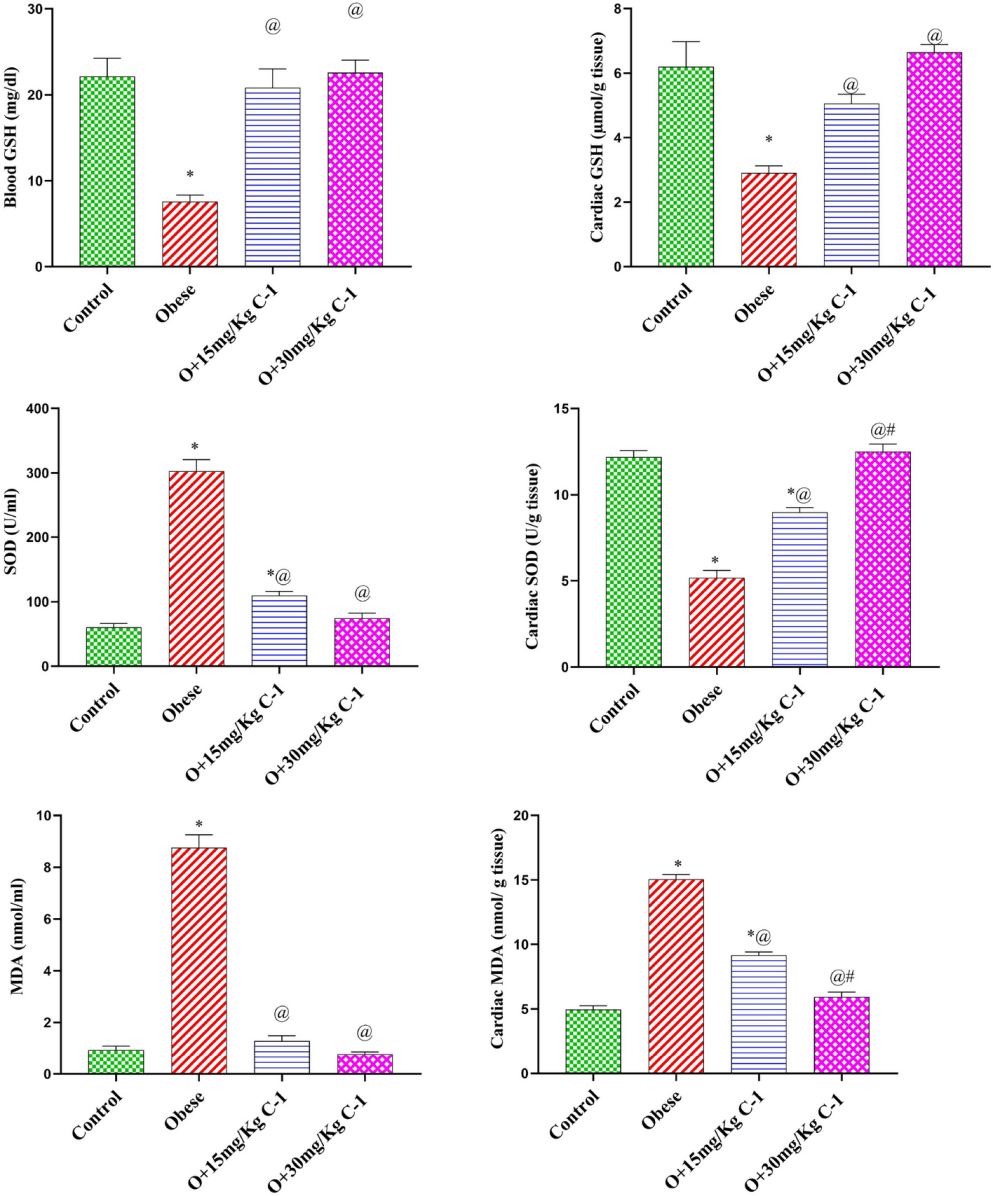
Effect of C-1 on oxidative stress parameters in plasma and cardiac tissue of group 2. Each bar represents the mean of 8 animals ± se. Statistical analysis was performed using one-way ANOVA followed by Tukey-Kramer multiple comparisons test. ($\star$ vs control group(group 1), @ vs obese group(group 2) and # vs Nano cerium oxide (15 mg/kg, group 3)) at $p<0.05$.

Group 2-evoked oxidative stress which manifested by a rise in the MDA content of the plasma and cardiac tissue by 9.3 and 3-fold, as well as a reduction in the cardiac or blood GSH by 65 and 84 % compared to the group 1 (Fig. 6). Moreover, cardiac SOD level decreased significantly in group 2 by 58 % ,while it elevated significantly in plasma by 53 %. Counter wise, treatment with the C-1 (groups 3 and 4) retracted the oxidative stress established by a rise in the GSH by about 3-fold in the plasma and by about 2-fold in the cardiac tissue, along with an increased cardiac SOD activity by 2.4-fold and about 8-fold compared to the group 2. Also, the C-1 (15 or 30 mg/kg) decreased the MDA level by 85 and 91 % in the plasma and by 39 and 61 % in the cardiac tissue compared to group 2. Cardiac or plasma GSH,SOD, and MDA content of group 4 did not change significantly relative to group 1 values

### Effect of C-1 on the Cardiac biomarker of obese rats in plasma

**Figure 7:**
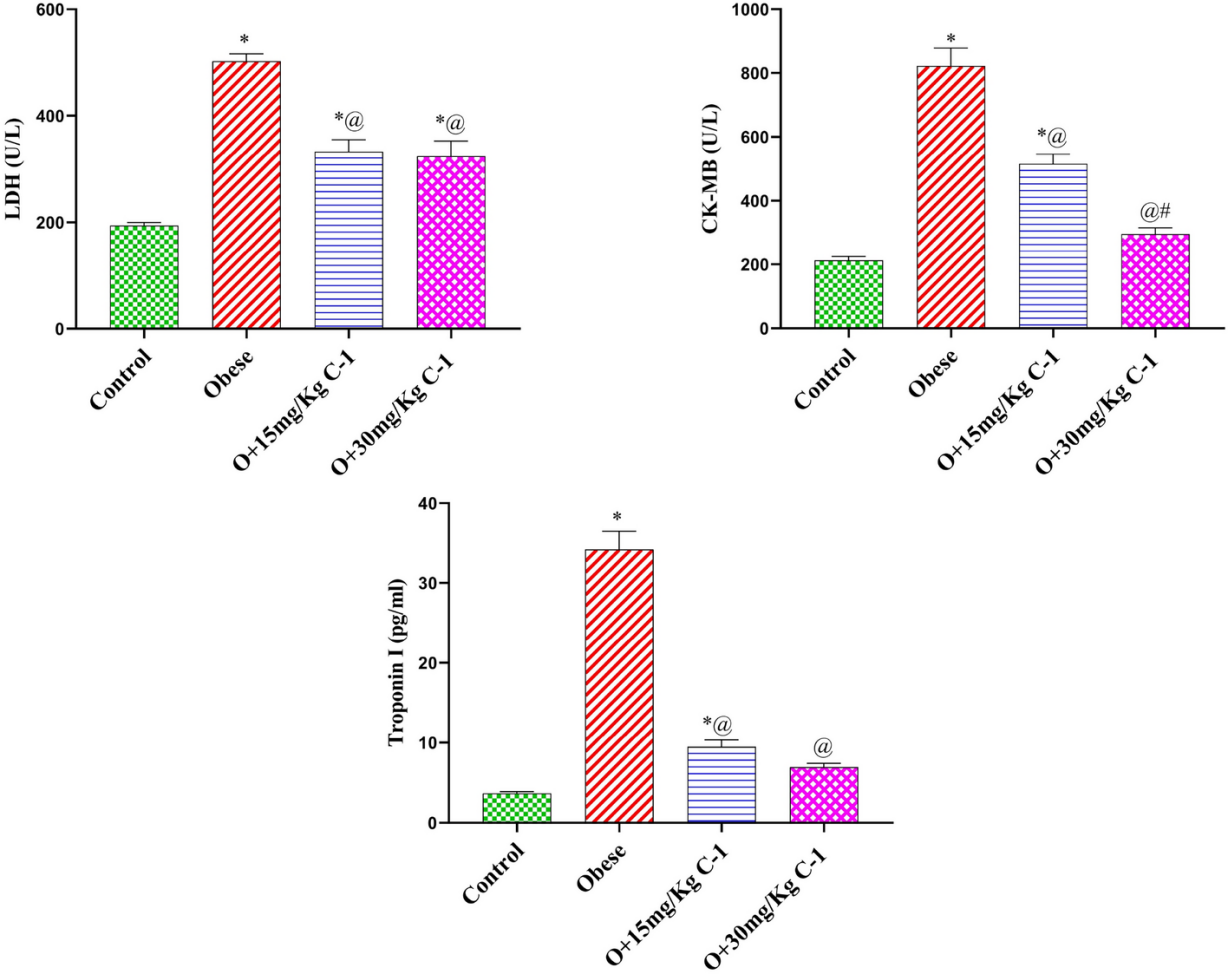
Effect of C-1 on cardiac biomarker parameters in plasma of obese rats. Each bar represents the mean of 8 animals ± se. Statistical analysis was performed using one-way ANOVA followed by Tukey-Kramer multiple comparisons test. ($\star$ vs control group(group 1), @ vs obese group(group 2) and # vs Nano cerium oxide (15 mg/kg, group 3)) at $p<0.05$.

Group 2 induced changes in cardiac biomarkers manifested by high activity of LDH CK-MB and troponin-1 by 3.8, 2.6 and 9.3-fold compared to the group 1 (Fig. 7). However, the treatment with C-1 (groups 3 and 4) showed lower LDH activity by 37, 64 and 72 % as well as lower CK-MB by 34, 36 % and 80 % than group 2. Plasma troponin, and CK-MB levels of group 4 did not change significantly relative to group 1 values

### Effect of C-1 insulin resistance parameters of obese rats

**Figure 8:**
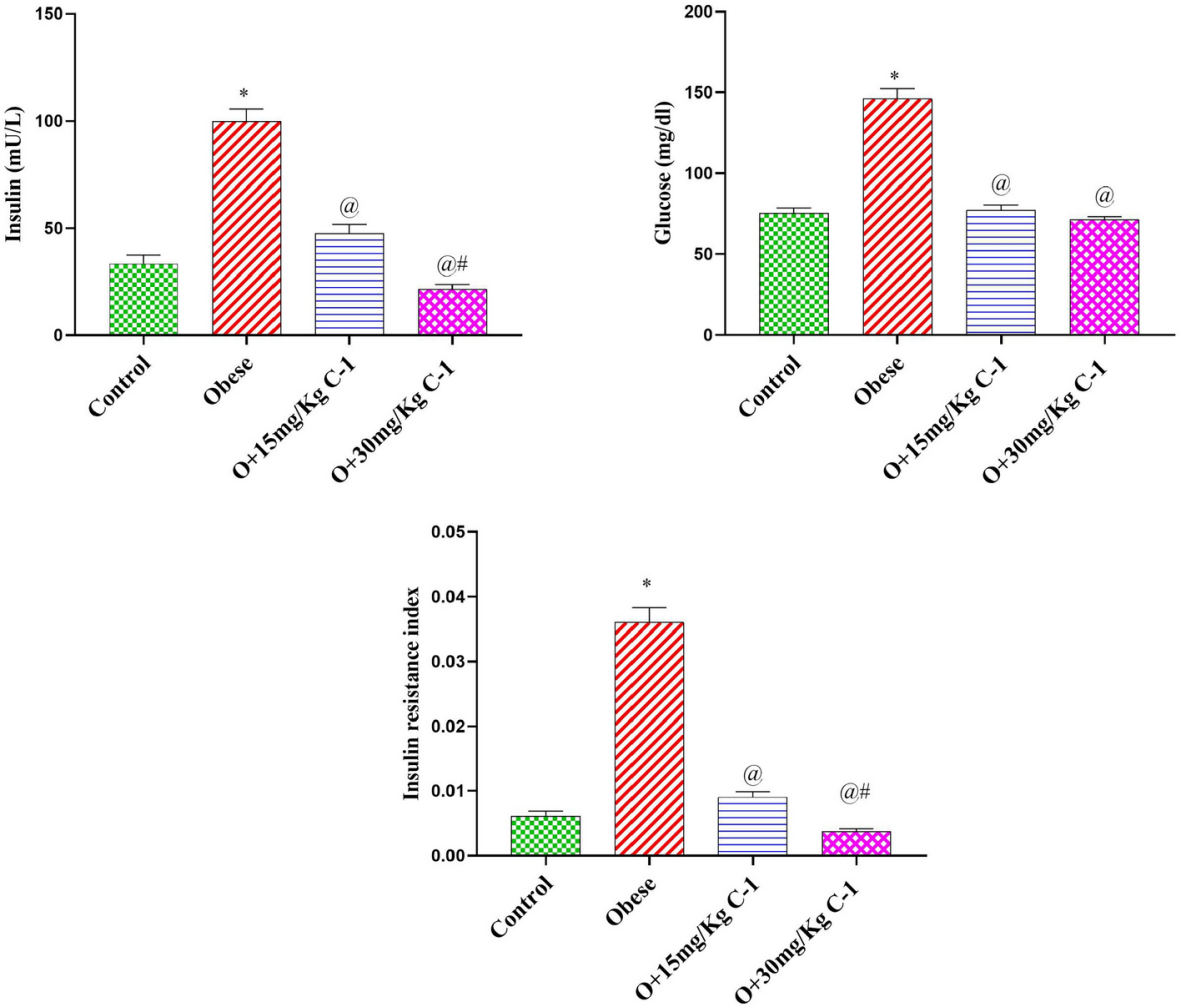
Effect of C-1 on insulin resistance parameters in plasma of obese rats.Each bar represents the mean of 8 animals ± se. Statistical analysis was performed using one-way ANOVA followed by Tukey-Kramer multiple comparisons test. ($\star$ vs control group(group 1), @ vs obese group(group 2) and # vs Nano cerium oxide (15 mg/kg, group 3)) at $p<0.05$.

As shown in Fig. 8, group 2 derangement in glycemic rats manifested by elevated glucose, insulin, and insulin resistance index by 3,1.9 and 8.6-fold compared to the group 1. Meanwhile, the C-1 (15 or 30 mg/kg) decreased the glucose, insulin, and insulin resistance index by 53, 47, and 75 % for group 3 and 78, 51, and 83 % for the group 4 compared to group 2. No significant difference was noted between groups 3,4 and group1 in all insulin resistance parameters.

### Effect of C-1 on the plasma inflammatory markers of obese rats

**Figure 9:**
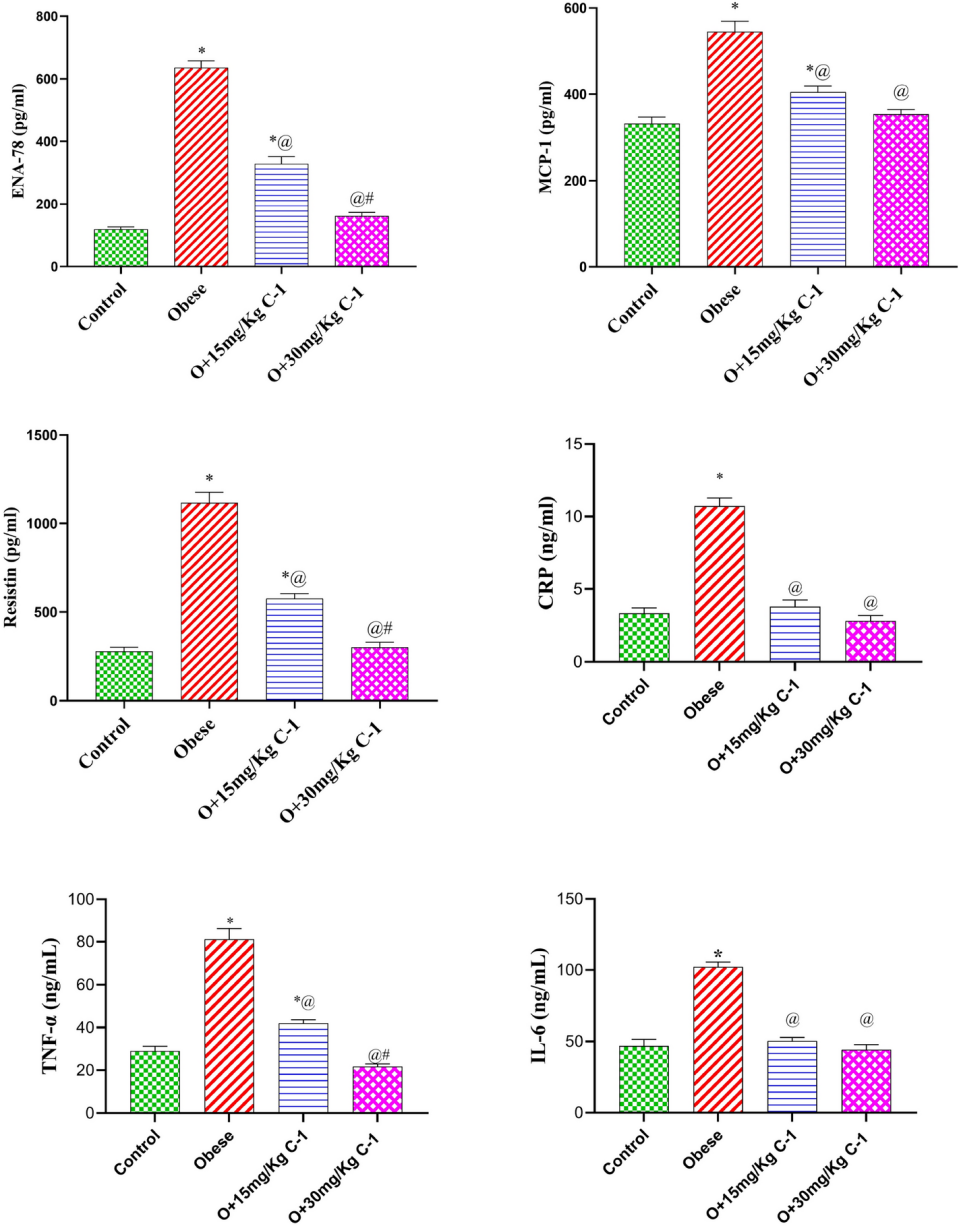
Effect of C-1 on inflammatory adipokines level of obese rats.Each bar represents the mean of 8 animals ± se. Statistical analysis was performed using one-way ANOVA followed by Tukey-Kramer multiple comparisons test. ($\star$ vs control group(group 1), @ vs obese group(group 2) and # vs Nano cerium oxide (15 mg/kg, group 3)) at $p<0.05$.

group 2 suffered from increased levels of ENA-78, MCP-1, and resistin, by 5.3, 1.6, and 4, compared to group 1 (Fig. 9). On the other hand, the administration of C-1 (15 or 30 mg/kg) countered this elevation by 48, 25, and 49 % for group 3 and by 75, 35, and 73 % for group 4 compared to group 2. Similarly, group 2 underwent an elevation in the content of CRP, TNF$\alpha$ and IL6 by 3.2, 2.8 and 2.1-fold compared to group 1. Counter wise, the administration of C-1 (15 or 30 mg/kg) reduced the cardiac CRP, TNF$\alpha$ and IL6 by 65, 48 and 51 % for group 3 and by 74, 73 and 57 % for the group 4 compared to group 2. The inflammatory adipokines level of group 4 did not change significantly relative to group 1.

### Histological study

**Figure 10:**
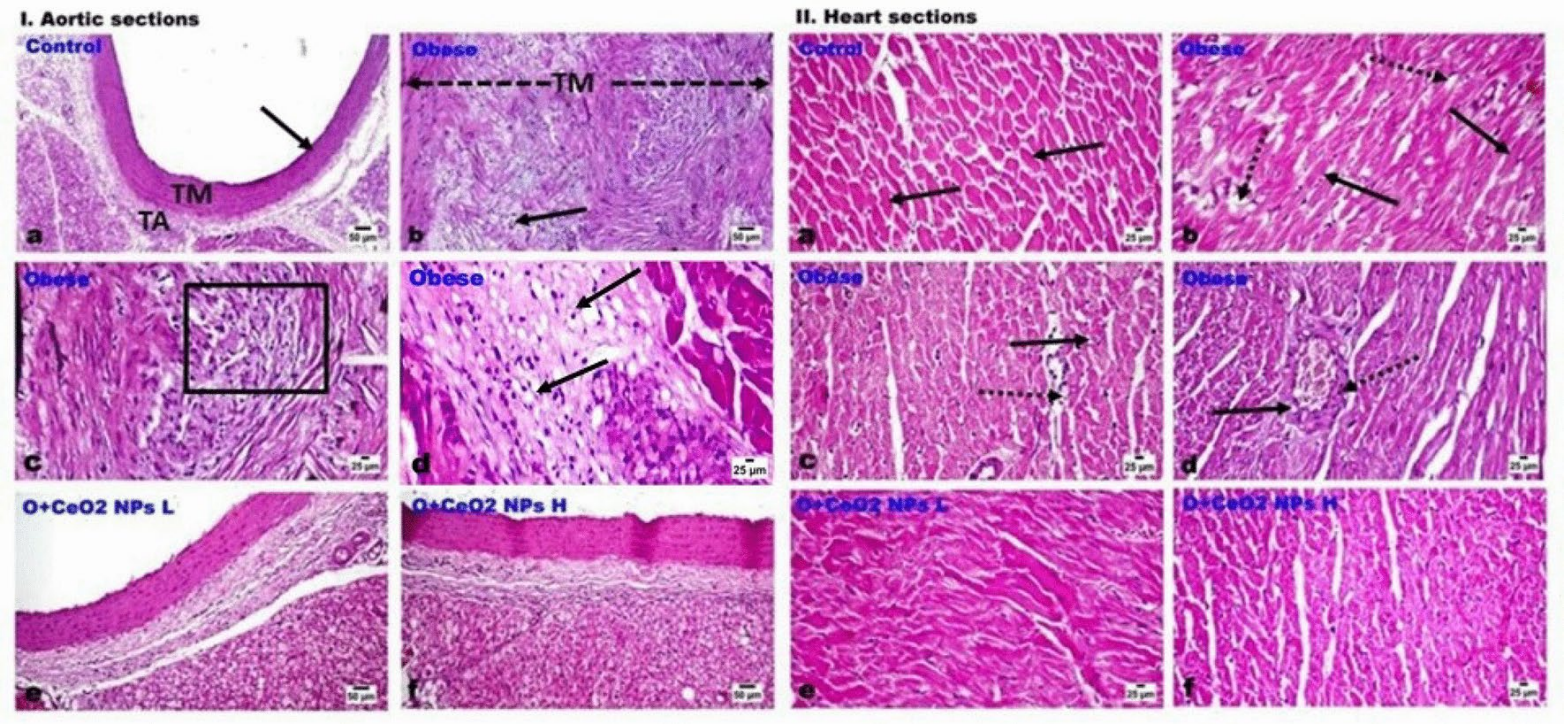
I and II Photomicrographs of H&E-stained sections of aorta and cardiac muscles. I (**a**) Abdominal aorta of control rat showing normal tunica intima (arrow), media (TM) and adventitia (TA). (**b**–**d**) aorta of obese rat showing severe thickening of TM with swelling and degeneration of its muscle fibers (arrow), splitting of atherosclerotic plaque by inflammatory cells, foamy macrophages, and free RBCs, (square) with many lipophages (foam cells) (arrow) in the medial layer. (**e** and **f**) C-1 treated rats showing significant improvement in aortic histological alterations particularly at the group 4 group (**f**) with only few inflammatory cells infiltrating the TM with variable degree of adventitial edema. II (**a**) cardiac muscles of control rat showing normal cardiomyocytes (arrow). (**b**–**d**) cardiac muscles of obese rat showing (**b**) marked myofibers’ vacuolar degeneration (dotted arrow) and scattered hypereosinophilia (arrow), (**c**) apoptotic cells (arrow), and increased inter-muscular fat (dotted arrow) (**d**) coronary vessel showing medial thickening (dotted arrow) and vacuolization (arrow). (**e** and **f**) C-1 treated rats at low and group 4 respectively, showing significant restoration of cardiomyocytes integrity with only mild degeneration and scattered muscular eosinophilia.

The abdominal aorta of control rats showed the normal histological structure of all its tunicae layers: intima (TI), media (TM) and tunica adventitia (TA) without any histological alterations (Fig. 10. Ia). In obese rats, their aortae displayed marked histological changes and marked thickening of their walls with prominent reduction in the elastic fibers in all tunicae. The intimal layer revealed irregularity; its endothelial linings showed swelling, cytoplasmic vacuolation, and focal desquamation. The TM was severely expanded and showed swelling and degeneration of its muscle fibers with their splitting by an obvious atherosclerotic plaque of mononuclear inflammatory cells, foamy macrophages, free RBCs, (Fig. 10. Ib) and Small foci of granulation tissue (Fig. 10. Ic). Many lipophages (foam cells) were evident (Fig. 10. Id) along the medial layer. The later alterations were conspicuously reduced with the administration of C-1 at low (Fig. 10. Ie) and high (Fig. 10 If) doses, particularly in the later dose group, which showed significant improvement in the histological changes. Only a few inflammatory cells were observed scattered and infiltrating the TM with variable degrees of adventitial edema with a profound reduction in the aortic tunicae thickening observed

**Figure 11:**
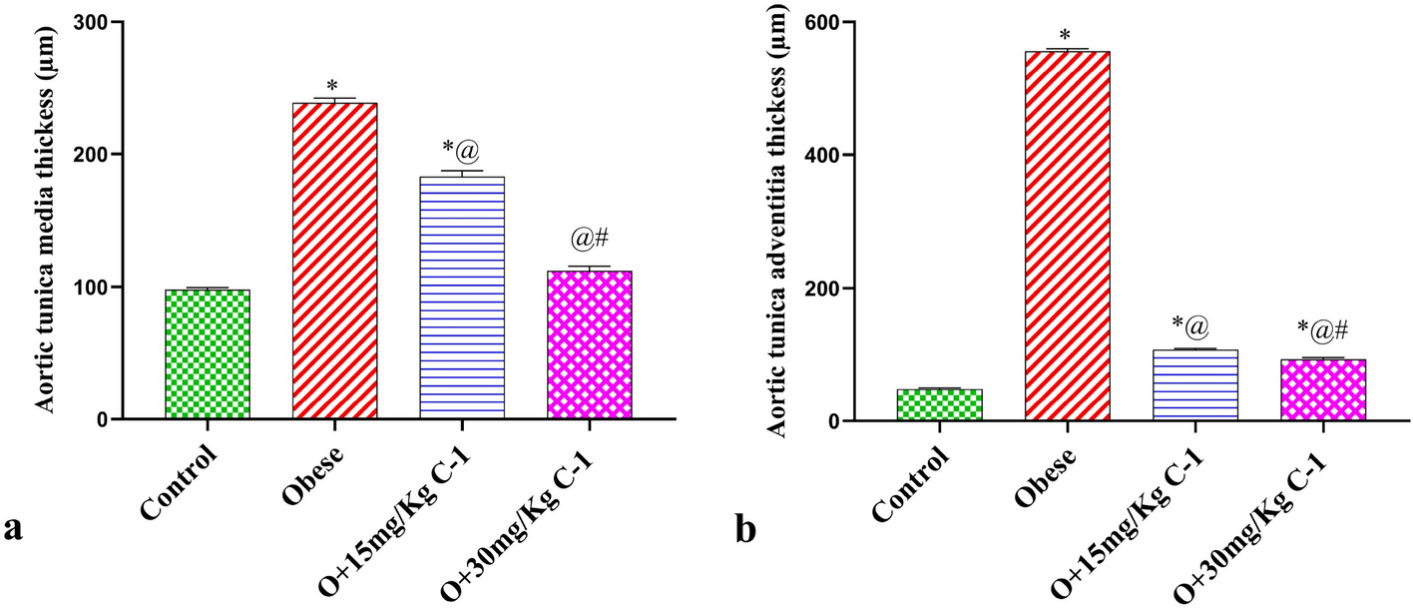
Marked thickening of the both tunicae media and adventitial of aorta of obese rats and significant dose-related reduction in that thickening in C-1 treated rats’ groups. Each bar represents the mean ± SE of 8 rats. *vs normal control group(group 1), @vs obese group(group 2), #vs group 3 (15 mg/kg C-1) at $p < 0.05$.

There is a notable increase in the thickness of both the tunica media and adventitia in the aorta of obese rats (Fig. 11a, b). However, a noteworthy and dose-dependent reduction in this thickening is observed in groups of rats treated with C-1 (Fig. 11a, b). The data indicates a clear relationship between the dosage of C-1 and the mitigation of aortic thickening in treated rats. Each bar in the representation signifies the mean value, providing a comprehensive view of the dosage-dependent impact of C-1 on reducing the observed thickening in both the tunica media and adventitia of the aorta in obese rats. This underscores the potential efficacy of C-1 in addressing vascular alterations associated with obesity.

Additionally, there was a significant restoration of the elastic lamellae to an almost control-like structure, particularly in the high-dose group. The examination of the cardiac muscles of control rats revealed normal histological structure, orientation, and striation (Figure 10. IIa). Examination of cardiac muscles of obese rats showed marked myofibers’ degenerative changes, which appeared as marked swelling, vacuolation, and scattered hypereosinophilia, (Fig. 10. IIb). Loss of striation was evident together with muscle fragmentation, granularity of the sarcoplasm, apoptotic cells, and increased inter-muscular fat was also noticed (Fig. 10. IIc). Infiltration of macrophages was seen sometimes in a focal manner phagocytosing the debris. The coronary vessels in the vicinity showed perivascular edema, medial thickening with vacuolization and edema (Fig. 10. IId) with sometimes focal fibrinoid necrosis. The administration of C-1 significantly restored the integrity of the cardiomyocytes in both the low (Fig. 10. IIe) and high (Fig. 10. IIf) dose groups, particularly with the later dose administration. Only, mild degenerative changes and scattered muscular eosinophilia and necrosis with an obvious restoration of the vascular pathology.

### Immunohistochemical stain for iNOS expression

**Figure 12:**
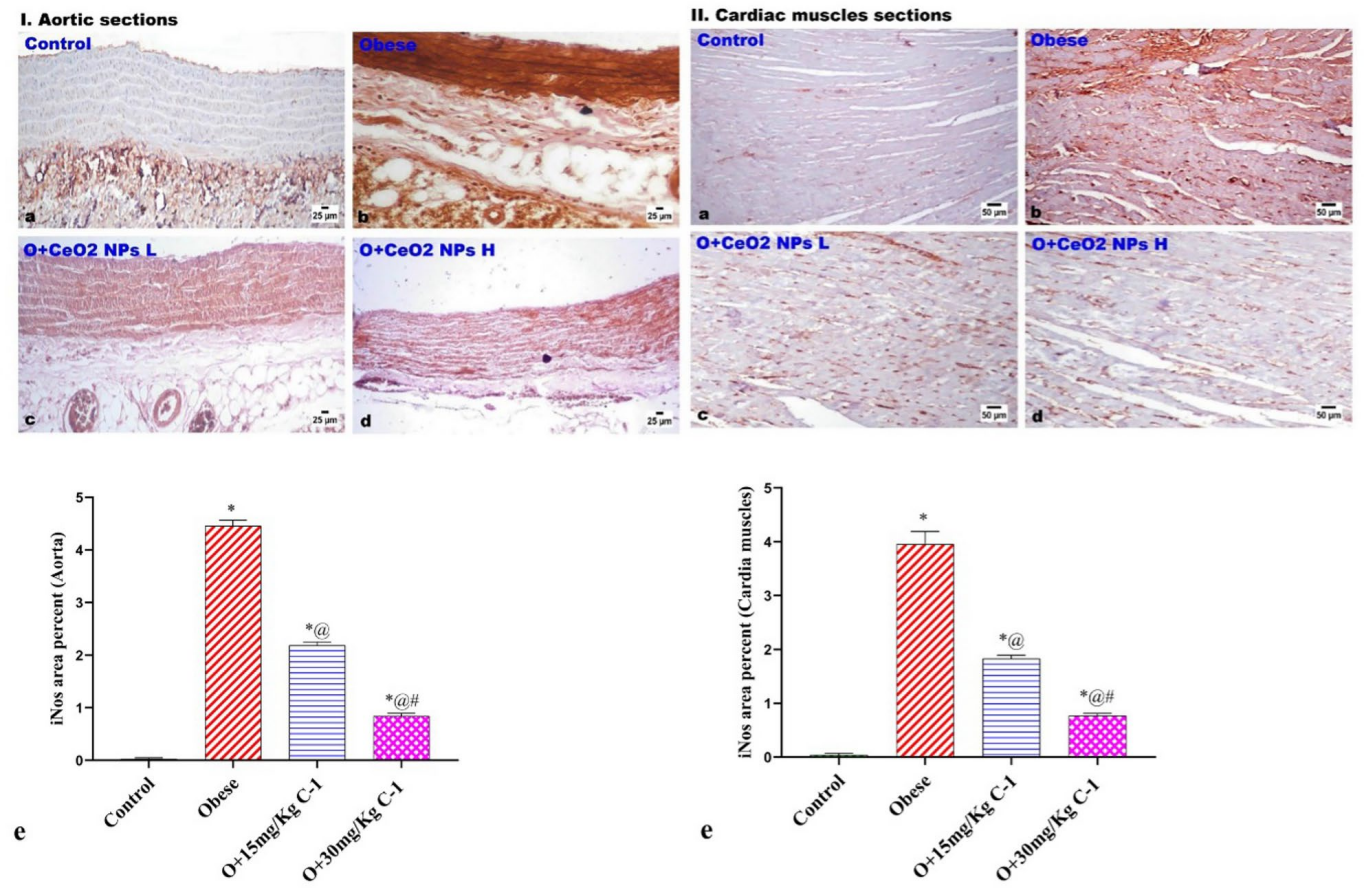
Photomicrographs of iNos immune-stained aortic and cardiac muscles of all groups. Obese rats (group 2) exhibited a significant increase in iNos expression. C-1 treated groups showing significant reduction in iNos expression. . Each bar represents the mean ± SE of 8 rats. *vs normal control group(group 1), @vs obese group(group 2), #vs C-1 ( 15 mg/kg) at $p < 0.05$.

The administration of C-1 resulted in significantly increased expression of iNOS in the aortic walls as well as in the cardiac muscles (Fig. 12. I & II) of obese model rats as presented in particularly in the group 4 administrated rats.

## Discussion

The C-1 XRD diffraction peaks at $28.52^{\circ }, 32.97^{\circ }, 47.40^{\circ }, 56.19^{\circ }, 58.94^{\circ }, 69.17^{\circ }, 76.52^{\circ }$, and $78.86^{\circ }$ may be indexed to (1 1 1), (2 0 0), (2 2 0), (3 1 1), (2 2 2), (4 0 0), (3 3 1), and (4 2 0) respectively for cubic structure of CeO$_{2}$ with space group F m -3 m (225) which was matched by XRD reference codes COD: 1562989. The cell parameters were calculated using the peak at $28.52^{\circ }$ which is indexed to add (1 1 1) plane. The calculated value (5.42 Å) is very close to that (5.41 Å) of COD: 1562989. The slightly broadning in peaks indicates small particles sizes. Using the modified sol-gel approach, cerium oxide was produced and designated as A-1 in one of our earlier studies^23^. Figure 1b: c and d illustrate how the peak broadening of the planes (1 1 1) and (2 0 0) of C-1 (FWHM: 0.40 for both) differs from that of A-1 (FWHM: 0.35 (1 1 1), and 0.30 (2 0 0))^23^. The crystallite size of C-1 was calculated using all peaks. The results indicate a decrease in average crystal size (20.74 nm) compared to that of A-1 (42.81 nm)^23^. This may be caused by using a modified sonication-sol-gel method . S$_{T}$ (12.73 m$^{2}$g$^{-}$) , and S$_{BET}$ (145.78 m$^{2}$) were calculated using adsorption isotherm of C-1 (Fig. 1g). This isotherm consists of three stages: $\hbox {P /P}_{o} < 0.15$, $0.15< \hbox {P/P}_{o} < 0.77$, and $\hbox {P/P}_{o} > 0.77$, which is similar to nonporous materials of type II with a mixed hysteresis of H1 and H3 type. pdn calculated using the BJH model (Fig.  1g), showing a peak at 10.38 nm . The morphology of the C-1 was investigated using TEM (Fig. 1e), which revealed its highly crystalline nature with regular particles. The HRTEM image shows clear (1 1 1), (2 0 0), and (2 2 0) lattice fringes, indicating that current nanoparticles are dominated by a truncated octahedral shape enclosed by the [1 1 1] and [1 0 0] facets.^27^. The Raman spectrum of C-1 did not show peaks around 300 cm^−1^ which indicate the absence of rearrangement oxygen atoms and the presence of oxygen in its fluorite lattice positions.^27^. The doublet (at 454.93 and 461.07 cm^−1^) and the peak at 1167.85 cm^−1^ were assigned to the vibration model (F_2g_) and the oxygen vacancies (O_v_) in CeO$_{2}$.^28,29^. The peaks at 553.68 and 582.22 cm^−1^ were attributed to oxygen vacancies^30,31^ , while that around 606.12 cm^−1^ was attributed to the presence of Ce^3+^^32^ or an oxygen vacancy created due to the movement of an oxygen atom into an octahedral interstitial position (a Frenkel anion pair)^33^. The average particle diameters obtained from TEM histogram data (21.09) are very similar to those calculated using the Debye-Scherrer Method (20.74 nm). The survey spectra XPS spectrum of C-1 is showmen in Fig. 1a. The spectrum shows three peaks at 902.45 eV, 533.99 eV, and 290.62 eV corresponding to Ce-3d (26.05 Atomic %), O-1s (73.95 Atomic %), and C-1s (0.00 Atomic %), respectively. The presence of C (C-1 s) which is found as a trace (0.00 Atomic %) may be due to the adsorption on the surface of the sample, as a residue or during sample preparation. The high-resolution (HR) O-1s XP spectrum is shown in Fig. 1a. The spectrum indicates the presence of three peaks at 530.85, 534.52, and 537.11 eV are associated with metal oxide, O$\gamma$ in water species, and water vapor phase.^23,34–36^. HR Ce-3d XPS spectrum is shown in Fig. 1a. The presence of ten peaks indicates the existence of both Ce^3+^ and Ce^4+^. These two states have distinct line shapes of their final states: Ce^4+^ = v + v^,,^ + v^,,,^ + u + u^,,^ + u^,,,^, and Ce^3+^= v_o_ + v^,^ + u_o_ + u^,^^37^. In these spectra, the peaks v_o_, v, v^,^, v^,,^, v^,,,^, u_o_, u, u^,^, u^,,^, u^,,,^ were found at 883.37, 886.58, 890.06, 893.24, 899.12, 902.28, 905.73, 910.70, 916.58, and 920.26, respectively. The binding energies u /v, u_o_ /v_o_ and u^,^/v^,^/u^,,^/v^,,^ are the result of O 2p and Ce 4 f screening level hydration corresponds to Ce: 3d^9^4f^4^O2p^4^, Ce: 3d^9^ 4f^2^ O 2p^4^, Ce: 3d^9^ 4f^1^ O 2p^5^ while u^,,,^/v^,,,^ results from 3d^9^ 4f^0^ O 2p^6^ final state^38^. The last peak (u^,,,^) is indicated as a fingerprint for +4 state^23^. The % Ce^4+^ can be calculated using the equation 3^23^. The % Ce^4+^ was found to be 57.01 %.

3 $$\begin{aligned} \% Ce^{4+} = \frac{\sum \hspace{5pt} of \hspace{5pt} v + v^{,,} + v^{,,,} + u + u^{,,} + u^{,,,} \hspace{5pt} peak \hspace{5pt} area* 100 }{Total \hspace{5pt} peak \hspace{5pt} area} = 57.01 \% & \end{aligned}$$

Obesity is characterized by adiposity posing potential risks to health. It is a significant risk factor for numerous diseases, notably CVDs. The measurement of high waist circumference (W.C.) in individuals with obesity can serve as an important predictor of CVD risk. This is attributed to the fact that W.C. serves as an indicator of abdominal body fat, which is closely linked to the development of cardiometabolic diseases. Monitoring waist circumference is therefore valuable in assessing the potential health implications of obesity, particularly its association with cardiovascular risk factors^39^. Our results elucidated that the treatment of overweight rats with C-1 reduced the BMI, weight and W.C. Indeed, studies have reported that weight loss in obese individuals can lead to improvements or prevention of many risk factors associated with CVD. Losing excess weight has been shown to positively impact various cardiovascular risk factors, including blood pressure, cholesterol levels, blood sugar control, inflammation, reduced strain on the heart and improved endothelial function^40^. Both BMI and W.C. were effective in predicting the risk of CVD. The improvement of anthropometric measures of obese rats by C-1 can be attributed to their excellent observed effect on a reduction of adipose tissue weight or to combating the decrease in adiponectin level that lowers body weight and boosts fatty acid oxidation by the muscle^41^

It is well known that weight loss in obese people is linked with a decline in oxidation markers, enhancement of antioxidant defenses, and reducion of pathological risk factors. Certainly, there is ample documentation supporting the notion that weight reduction yields several positive effects on health. Weight loss has been well-documented to result in the following beneficial changes such as decreased oxidation markers, increased antioxidant defenses and reduction in obesity-associated pathological risk factors^42^.

Our study found that oxidative stress (OS) went down a lot when C-1 was injected into overweight rats. The study found that C-1 can help overweight people lower OS. MDA level is a sign of lipid peroxidation. In other word, C-1 can reduce the fat oxidation. Additionally, the antioxidant GSH levels went up in both plasma and heart tissue when C-1 was present. This suggests that the body’s antioxidant defenses got stronger. Also, C-1 stopped SOD activity from going down in heart tissue and raised SOD activity in plasma, which suggests that they are good for antioxidant enzymes. It’s important to remember that OS markers are strongly linked to body mass index (BMI). These results back up earlier research that suggested C-1 might be able to help treat OS-related problems in obese people^43^. Based on our research, we found that BMI and GSH levels were linked negatively . What this means is that having a higher BMI may make antioxidant defenses less strong. But there was a clear link between BMI and both SOD activity and MDA levels, which shows that lipids are being peroxidized. It is obvious that having a higher BMI is linked to having more OS. In the study’s conclusion, it was said that C-1 protects against OS and is safe to use in medicine. This adds to what other people have done^44^. CeNPs can switch between Ce$^{3+}$ and Ce$^{4+}$ oxidation states, which is what makes this happen. They are a good choice for ROS mediation because of this. CeNPs are said to protect against OS, which may be because they can switch between the Ce$^{3+}$ and Ce$^{4+}$ states. This is one of the special things about CeNPs that makes them perfect for neutralizing ROS. The Ce$^{3+}$ and Ce$^{4+}$ nanoparticles can effectively neutralize ROS because they move back and forth quickly. This lowers OS in general. As El-Seidy et al. explain, this natural property suggests that cerium nanoparticles might be useful for treating diseases linked to OS. This means that they could be used in medicine^45^.

Celardo et al. say that cerium oxide nanoparticles also have properties like catalase and SOD^46^. C-1 also kept cardiomyocytes whole, which slowed down the changes that happened in the heart muscles of overweight rats. The main reason for this is likely that they lowered MDA levels in the heart and raised GSH and SOD levels. The defense role in the cells, with the aid of GSH, helps them get rid of free radicals. It is important to protect the myocardium and get rid of any extra ROS. A lot of injurious things can happen to the heart, but the GSH system is an important part of protection against them. To find ways to lower the health problems linked to OS in the heart, it is important to know how the GSH system works and keep it in good shape.^47^

Also, C-1 can lower the amount of iron that builds up in both fat tissue and heart tissue. According to Sachinidis et al., this drop in iron buildup is important because it is linked to metabolic syndrome and heart disease^48^. Another possible source of OS in obesity was the elevation of cardiac and adipose iron content observed in the present work. The link between iron overload and OS associated with metabolic dysfunction, type 2 diabetes, and CVD was reported^48,49^. It looks like C-1 protects the heart muscle cells in overweight rats by either stopping the production of leptin or raising the levels of adiponectin. Hyperleptinemia was shown to make rat cardiomyocytes make more inflammatory mediators, which led to individual cardiac cells getting bigger that may cause cardiomegaly , as shown by Ghantous and his coworker^50^. Adiponectin, on the other hand, is a hormone that helps the heart stay healthy by lowering inflammation, OS, and left ventricular and vascular hypertrophy^19,50^. It was found that C-1 could stop the damage that cigarette smoke extract causes to cardiomyocytes by stopping the production of ROS, starting the NF-$\kappa$B cascade, increasing the expression of inflammatory genes, and using up all the antioxidants in the body. It was discovered that C-1 lowers OS and inflammation in the myocardium, which slows the development of heart failure. This is most likely because they are self-regenerating antioxidants^51^.

OS is a central player in the development of complications associated with obesity, and its origins in this context are multifaceted. Hyperglycemia, a consequence of insulin resistance often present in obesity, contributes to OS by fostering the production of ROS. Concurrently, dyslipidemia, characterized by elevated lipid levels, promotes lipid peroxidation and further amplifies oxidative damage. Chronic low-grade inflammation, a hallmark of obesity, stimulates the release of inflammatory molecules, contributing to ROS production and sustained OS. This intricate interplay among hyperglycemia, elevated lipid levels, and chronic inflammation creates a cycle that not only exacerbates metabolic dysfunction but also fosters OS, underlining the significance of addressing these factors to mitigate obesity-related complications^52^ and hyperleptinemia^53^.The BMI and OS indicators have been shown to have a strong direct correlation^43^. It appears that C-1 reduced OS in obese rats directly through their antioxidant property or indirectly via lowering BMI, lipids, inflammatory markers, and leptin levels besides enhancement of adiponectin level. It appears that obesity increases the risk of cardiovascular disease and early atherosclerotic changes via many factors, such as OS, hyperlipidemia, hyperglycemia, hyperleptinemia, I.R. (inflammatory response), cytokines, and inflammation^54,55^. Our results showed some pathological changes in the aorta of obese rats represented by irregularity of the intimal layer, swelling of its endothelial linings, and cytoplasmic vacuolation. The tunica media was severely expanded and showed swelling and degeneration of its muscle fibers with their splitting by atherosclerotic plaque of mononuclear inflammatory cells and foamy macrophages. The increase of OS is a risk factor for the development of atherosclerosis. The interaction of ROS and nitric oxide is crucial in the development of endothelial dysfunction through generation of peroxynitrite. The increase in ROS production initiates cascade of events that aggravate vascular endothelial damage via elevation of oxidation of low-density lipoprotein in atherosclerotic lesions, and increased expression of adhesion molecules, which results in macrophage migration and the formation of lipid-laden macrophages^52^The proatherogenic role of oxidized LDL may be performed through chemotactic and proliferating actions on monocytes/macrophages, exciting their transformation into foam cells, through stimulation of smooth muscle cells (SMCs) recruitment and proliferation in the tunica intima, or via evoking endothelial cells, SMCs, and macrophages apoptosis^56^. On the other hand, HDL promotes reverse cholesterol transport and modulates inflammation. HDL-cholesterol levels are inversely correlated with the risk of cardiovascular events^57^.

It appears that C-1 mitigates the increase in aorta thickening of obese rats by affecting insulin resistance, adipokines hormones (leptin, adiponectin, resistin) and MCP-1,a chemokine Our results indicate that the treatment of obese rats with C-1 reduced insulin resistance which can contribute to endothelial dysfunction and atherosclerosis via induction of hyperglycemia along with dyslipidemia that causes OS and inflammatory response^58^. It was suggested that C-1 could have a potential insulin-sensitizing effect specifically on adipose tissue and skeletal muscle as related to mitochondrial function^59^.

C-1 exhibits a protective effect against the elevation of leptin, resistin, MCP-1, and ENA-78 levels observed in obese rats. Elevated levels of leptin, resistin, MCP-1, and ENA-78 are associated with various aspects of obesity-related complications. Specifically, hyperleptinemia is known to stimulate monocyte entry into blood vessels, transform macrophages into foam cells, induce the proliferation of vascular smooth muscle cells, and trigger the secretion of atherosclerotic cytokines. Likewise, C-1 treatment led to a decrease in the TG/HDL ratio, which may indicate the lowering of add risk of cardiovascular disease . These collective processes contribute to the development of atherosclerosis. C-1 mitigated the increase in these adipokines, and chemokines may play a role in preventing or ameliorating the cascade of events associated with hyperleptinemia and its contribution to atherosclerosis in the context of obesity^60^. It was concluded that higher serum resistin (Inflammatory marker of atherosclerosis) concentration is associated with CVDs^61^. It was reported that resistin is involved in the pathogens of inflammation, insulin resistance, atherosclerosis, and atherogenic dyslipidemia. Moreover, resistin induces the expression of adhesion molecules and inflammatory markers like monocyte chemoattractant protein-1 (MCP-1), TNF$\alpha$, and IL6^62^

Without a doubt, MCP-1 is closely connected to problems with blood vessels. Over time, this makes atherosclerosis more developed.. Monocytes can move into the area around blood vessels with the help of MCP-1. There, they associate with oxidized low-density lipoprotein (ox-LDL) and change into foam cells. Fat deposits form because of this process. These deposits are the first sign that plaque is building up in blood vessels. The powerful chemotactic drug MCP-1 makes it easier for monocytes, T cells, and basophils to move from the bloodstream to damaged tissues. This makes swelling worse and raises the risk of getting heart disease. The fact that MCP-1 is important at different stages shows how important it is to understand the heart and blood vessel problems that come from being overweight. MCP-1’s main job is to make it easier for more monocytes to enter the body, which leads to more inflammation and the formation of foamy cells. A very important part of this process is speeding up the stiffening of the arteries. Consequently, it becomes a captivating topic to study in order to address issues pertaining to excessive weight and blood vessels^63^. There have been findings that indicate an increase in MCP-1 levels may result in I.R.^64^. Similarly, a number of other associations have been made between ENA-78 and obesity, inflammation, and insulin resistance^65^.

C-1, on the other hand, increased adiponectin levels in obese rats, which is important for enhancing insulin sensitivity and decreasing metabolic abnormalities or inflammation^66^. Here, the treatment of obese rats with C-1 alleviated the significant increase in Pro-inflammatory Cytokines, which may be considered another mechanism of C-1 to preserve the aorta. It was reported that C-1 exhibited anti-inflammatory properties^67^. Obesity is linked with low-grade inflammation that is considered a vital risk factor for developing CVDs and metabolic syndrome via increased inflammatory mediators such as IL6 , CRP and TNF$\alpha$ , followed by vascular and endothelial dysfunction^68^. Furthermore, it is thought that the link between inflammation caused by obesity and endothelial dysfunction is the generation of cytokines linked with inflammation.

The treatment of obese rats with C-1 led to a significant decrease in iNOS expression in the heart and blood vessel walls. On the other hand, iNOS expression was much higher in obese positive control rats . It has been linked to the production of a harmful chemical called peroxynitrite when iNOS levels rise. Because of this, bad things happen inside our bodies, like lipids breaking down, which hurts blood vessels and leads to the formation of plaques that can narrow arteries. (C-1) have the potential to mitigate cardiovascular complications associated with obesity by inhibiting iNOS and reducing peroxynitrite concentrations. (C-1) have the ability to decrease the expression of iNOS and alleviate associated stress^69^.

Furthermore, there is a direct link between producing more peroxynitrite and the onset of heart failure. This takes place when there is a moderate infiltration of inflammatory cells leading to cardiomyopathy (the heart muscle becomes thicker, bigger, and more stretched)^70^.Research has shown that iNOS is involved in several health issues associated with inflammation and I.R.^71^. For example, it was previously shown that iNOS can make superoxide not related to NO^72^.The same thing happens with superoxide and hydrogen peroxide, both of which are harmful^73^.The observed alteration in iNOS expression by (C-1) indicates a possible mechanism by which these nanoparticles may provide protection against cardiovascular problems associated with obesity. This finding suggests a promising direction for future research on the therapeutic uses of these nanoparticles.

## Conclusion

The modified synthesis method led to the formation of smaller particle size (20.74) than that reported in our previously work (42.81 nm). The XRD patterns of C-1 indicates its cubic structure with space group F m -3 m (225) which was matched by code id 1562989 from COD. The Raman spectrum of CeO$_{2}$ indicate the absence of rearrangement oxygen atoms, the presence of oxygen in its fluorite lattice positions, and the oxygen vacancies in CeO$_{2}$ and the Ce vibration model (F_2g_). The presence of ten peaks in the high-resolution Ce-3d XP spectrum indicates the existence of both Ce^3+^ and Ce^4+^. Obesity with hyperglycemia, dyslipidemia, iron overload, over-expression of iNOS, and insulin resistance have increased OS, which may be associated with adipokine abnormalities and CVDs. The reduction of OS and adipokine abnormalities by (C-1) may serve as a target for the treatment of CVDs in obesity, particularly atherosclerosis. Moreover, the weight loss and a decrease in BMI of obese rats given (C-1), along with reduction of adipose tissue weight, may contribute to mitigating cardiovascular disease-associated- obesity. C-1 might be promoted as a novel approach to control CVDs. The antioxidative properties of (C-1) may have the potential to serve as a treatment for a broad spectrum of inflammatory diseases. (C-1) showed therapeutic efficacy on atherosclerosis and cardiac muscle abnormalities associated with obese rats, probably due to their antioxidant and anti-inflammatory properties that lead to lowering OS.

## Data Availability

Data is provided within the manuscript.

## References

[1] Powell-Wiley, T. M. et al. Obesity and cardiovascular disease: A scientific statement from the American Heart Association. *Circulation* **143**, e984–e1010 (2021). 33882682 10.1161/CIR.0000000000000973 PMC8493650

[2] Abdelkader, N. F., Elbaset, M. A., Moustafa, P. E. & Ibrahim, S. M. Empagliflozin mitigates type 2 diabetes-associated peripheral neuropathy: A glucose-independent effect through ampk signaling. *Arch. Pharm. Res.* **45**, 475–493 (2022). 35767208 10.1007/s12272-022-01391-5 PMC9325846

[3] Savage, D. B., Petersen, K. F. & Shulman, G. I. Mechanisms of insulin resistance in humans and possible links with inflammation. *Hypertension* **45**, 828–833 (2005). 15824195 10.1161/01.HYP.0000163475.04421.e4

[4] Trayhurn, P. The biology of obesity. *Proc. Nutr. Soc.* **64**, 31–38 (2005). 15877920 10.1079/pns2004406

[5] Wilcox, G. Insulin and insulin resistance. *Clin. Biochem. Rev.* **26**, 19–39 (2005). 16278749 PMC1204764

[6] Wang, C. C. L., Gurevich, I. & Draznin, B. Insulin affects vascular smooth muscle cell phenotype and migration via distinct signaling pathways. *Diabetes* **52**, 2562–2569 (2003). 14514641 10.2337/diabetes.52.10.2562

[7] Abdelbaset, M., Safar, M. M., Mahmoud, S. S., Negm, S. A. & Agha, A. M. Red yeast rice and coenzyme q10 as safe alternatives to surmount atorvastatin-induced myopathy in hyperlipidemic rats. *Can. J. Physiol. Pharmacol.* **92**, 481–489 (2014). 24896301 10.1139/cjpp-2013-0430

[8] Zhao, S., Kusminski, C. M. & Scherer, P. E. Adiponectin, leptin and cardiovascular disorders. *Circ. Res.* **128**, 136–149 (2021). 33411633 10.1161/CIRCRESAHA.120.314458 PMC7799441

[9] Elseidy, A. M. et al. Zinc oxide nanoparticles characterization and therapeutic evaluation on high fat/sucrose diet induced-obesity. *Egypt. J. Chem.* **65**, 497–511 (2022).

[10] Salekeen, R. et al. Lipid oxidation in pathophysiology of atherosclerosis: Current understanding and therapeutic strategies. *Int. J. Cardiol. Cardiovasc. Risk Prev.* **14**, 200143 (2022). 36060286 10.1016/j.ijcrp.2022.200143 PMC9434419

[11] Stanimirovic, J. et al. A high fat diet induces sex-specific differences in hepatic lipid metabolism and nitrite/nitrate in rats. *Nitric Oxide Biol. Chem.* **54**, 51–9 (2016). 10.1016/j.niox.2016.02.007 26924725

[12] Feng, B. et al. Silymarin alleviates hepatic oxidative stress and protects against metabolic disorders in high-fat diet-fed mice. *Free Radical Res.* **50**, 314–327 (2016). 26758315 10.3109/10715762.2015.1116689

[13] Saleh, D. et al. Omega-3 fatty acids ameliorate doxorubicin-induced cardiorenal toxicity: In-vivo regulation of oxidative stress, apoptosis and renal nox4, and in-vitro preservation of the cytotoxic efficacy. *PLoS ONE* **15**, e0242175 (2020). 33180794 10.1371/journal.pone.0242175 PMC7660507

[14] Anavi, S. & Tirosh, O. inos as a metabolic enzyme under stress conditions. *Free Radical Biol. Med.* **146**, 16–35 (2020). 31672462 10.1016/j.freeradbiomed.2019.10.411

[15] Cyr, A. R., Huckaby, V. L., Shiva, S. S. & Zuckerbraun, B. S. Nitric oxide and endothelial dysfunction. *Crit. Care Clin.* **36**, 307–321 (2020). 32172815 10.1016/j.ccc.2019.12.009 PMC9015729

[16] Eilenberger, C. et al. Cytotoxicity, retention, and anti-inflammatory effects of a CeO nanoparticle-based supramolecular complex in a 3d liver cell culture model. *ACS Pharmacol. Transl. Sci.* **4**, 101–106 (2021). 33615164 10.1021/acsptsci.0c00170 PMC7887746

[17] Tisi, A. et al. Antioxidant properties of cerium oxide nanoparticles prevent retinal neovascular alterations in vitro and in vivo. *Antioxidants* **11**, 1133 (2022). 35740031 10.3390/antiox11061133 PMC9220105

[18] Chen, S. et al. Cerium oxide nanoparticles protect endothelial cells from apoptosis induced by oxidative stress. *Biol. Trace Elem. Res.* **154**, 156–166 (2013). 23740524 10.1007/s12011-013-9678-8

[19] Niu, J., Wang, K. & Kolattukudy, P. E. Cerium oxide nanoparticles inhibits oxidative stress and nuclear factor-B activation in h9c2 cardiomyocytes exposed to cigarette smoke extract. *J. Pharmacol. Exp. Ther.* **338**, 53–61 (2011). 21464334 10.1124/jpet.111.179978 PMC3126650

[20] Robinson, R. D., Spanier, J. E., Zhang, F., Chan, S.-W. & Herman, I. P. Visible thermal emission from sub-band-gap laser excited cerium dioxide particles. *J. Appl. Phys.* **92**, 1936–1941 (2002).

[21] Xu, C. & Qu, X. Cerium oxide nanoparticle: A remarkably versatile rare earth nanomaterial for biological applications. *NPG Asia Mater.* **6**, e90–e90 (2014).

[22] Bashandy, S. A. E. et al. Zinc nanoparticles ameliorated obesity-induced cardiovascular disease: Role of metabolic syndrome and iron overload. *Sci. Rep.* **13**, 16010 (2023). 37749096 10.1038/s41598-023-42550-y PMC10519991

[23] El-Seidy, A. M., Elbaset, M. A., Ibrahim, F. A., Abdelmottaleb Moussa, S. A. & Bashandy, S. A. Nano cerium oxide and cerium/zinc nanocomposites characterization and therapeutic role in combating obesity via controlling oxidative stress and insulin resistance in rat model. *J. Trace Elem. Med Biol.* **80**, 127312 (2023). 37804595 10.1016/j.jtemb.2023.127312

[24] Moridi, H. et al. Protective effect of cerium oxide nanoparticle on sperm quality and oxidative damage in malathion-induced testicular toxicity in rats: An experimental study. *Int. J. Reprod. Biomed.* **16**, 261–266 (2018). 29942934 PMC6004590

[25] Imeryuz, N. et al. Iron preloading aggravates nutritional steatohepatitis in rats by increasing apoptotic cell death. *J. Hepatol.* **47**, 851–859 (2007). 17825453 10.1016/j.jhep.2007.06.018

[26] Bancroft, J. & Gamble, M. *Bancroft’s theory and practice of histological techniques* (Elsevier, 2019).

[27] Ramli, A., Khairul Anuar, N. A. S. I., Bakhtiar, N. A. A., Mohamad Yunus, N. & Mohamed, A. R. Direct oxidation of hibiscus cannabinus stalks to vanillin using CeO nanostructure catalysts. *Molecules* **28**, 4963 (2023). 37446622 10.3390/molecules28134963 PMC10343839

[28] He, L. et al. Morphology-dependent catalytic activity of Ru/CeO in dry reforming of methane. *Molecules* **24**, 526 (2019). 30717097 10.3390/molecules24030526 PMC6385116

[29] Pathak, V. et al. Synthesis, characterization and applications of cubic fluorite cerium oxide nanoparticles: A comprehensive study. *Results Surfaces Interfaces* **11**, 100111 (2023).

[30] Li, S. et al. Europium-doped Ceria nanowires as anode for solid oxide fuel cells. *Front. Chem.* **8**, 348 (2020). 32523935 10.3389/fchem.2020.00348 PMC7261932

[31] Sartoretti, E. et al. In situ Raman analyses of the soot oxidation reaction over nanostructured ceria-based catalysts. *Sci. Rep.* **9**, 3875 (2019). 30846727 10.1038/s41598-019-39105-5 PMC6405916

[32] Filtschew, A., Hofmann, K. & Hess, C. Ceria and its defect structure: New insights from a combined spectroscopic approach. *J. Phys. Chem. C* **120**, 6694–6703 (2016).

[33] Dosa, M. et al. Novel Mn-Cu-containing CeO nanopolyhedra for the oxidation of CO and diesel soot: Effect of dopants on the nanostructure and catalytic activity. *Catal. Lett.* **148**, 298–311 (2018).

[34] Rojas, J., Toro-Gonzalez, M., Molina-Higgins, M. & Castano, C. Facile radiolytic synthesis of ruthenium nanoparticles on graphene oxide and carbon nanotubes. *Mater. Sci. Eng. B* **205**, 28–35 (2016).

[35] Qiu, J. et al. Catalytic activity, selectivity, and stability of co-precipitation synthesized Mn-Ce mixed oxides for the oxidation of 1,2-dichlorobenzene. *Environ. Sci. Pollut. Res.* **28**, 65416–65427 (2021). 10.1007/s11356-021-15016-9 34319524

[36] Mazzotta, E., Rella, S., Turco, A. & Malitesta, C. XPS in development of chemical sensors. *RSC Adv.* **5**, 83164–83186 (2015).

[37] Isaacs, M. A. et al. XPS surface analysis of ceria-based materials: Experimental methods and considerations. *Appl. Surface Sci. Adv.* **18**, 100469 (2023).

[38] Bêche, E., Charvin, P., Perarnau, D., Abanades, S. & Flamant, G. Ce 3d XPS investigation of cerium oxides and mixed cerium oxide (). *Surf. Interface Anal.* **40**, 264–267 (2008).

[39] Piché, M.-E., Poirier, P., Lemieux, I. & Després, J.-P. Overview of epidemiology and contribution of obesity and body fat distribution to cardiovascular disease: An update. *Prog. Cardiovasc. Dis.* **61**, 103–113 (2018). 29964067 10.1016/j.pcad.2018.06.004

[40] Sjöström, L. et al. Lifestyle, diabetes, and cardiovascular risk factors 10 years after bariatric surgery. *N. Engl. J. Med.* **351**, 2683–2693 (2004). 15616203 10.1056/NEJMoa035622

[41] Fruebis, J. et al. Proteolytic cleavage product of 30-kda adipocyte complement-related protein increases fatty acid oxidation in muscle and causes weight loss in mice. *Proc. Natl. Acad. Sci. U.S.A.* **98**, 2005–2010 (2001). 11172066 10.1073/pnas.041591798 PMC29372

[42] Sofi, F., Abbate, R., Gensini, G. F. & Casini, A. Accruing evidence on benefits of adherence to the mediterranean diet on health: An updated systematic review and meta-analysis. *Am. J. Clin. Nutr.* **92**, 1189–1196 (2010). 20810976 10.3945/ajcn.2010.29673

[43] Vincent, H. K. & Taylor, A. G. Biomarkers and potential mechanisms of obesity-induced oxidant stress in humans. *Int. J. Obes.* **30**, 400–418 (2006). 10.1038/sj.ijo.0803177 16302012

[44] Khorrami, M. B. et al. Antioxidant and toxicity studies of biosynthesized cerium oxide nanoparticles in rats. *Int. J. Nanomed.* **14**, 2915–2926 (2019). 10.2147/IJN.S194192 PMC6487897 31114200

[45] El-Seidy, A. M., Elbaset, M. A., Ibrahim, F. A., Abdelmottaleb Moussa, S. A. & Bashandy, S. A. Nano cerium oxide and cerium/zinc nanocomposites characterization and therapeutic role in combating obesity via controlling oxidative stress and insulin resistance in rat model. *J. Trace Elem. Med Biol.* **80**, 127312 (2023). 37804595 10.1016/j.jtemb.2023.127312

[46] Celardo, I., Pedersen, J. Z., Traversa, E. & Ghibelli, L. Pharmacological potential of cerium oxide nanoparticles. *Nanoscale* **3**, 1411 (2011). 21369578 10.1039/c0nr00875c

[47] Tan, M. et al. Glutathione system enhancement for cardiac protection: Pharmacological options against oxidative stress and ferroptosis. *Cell Death Disease* **14**, 131 (2023). 36792890 10.1038/s41419-023-05645-y PMC9932120

[48] Sachinidis, A. et al. Dysmetabolic iron overload in metabolic syndrome. *Curr. Pharm. Des.* **26**, 1019–1024 (2020). 32000639 10.2174/1381612826666200130090703

[49] Bashandy, S. A. E. et al. Zinc nanoparticles ameliorated obesity-induced cardiovascular disease: Role of metabolic syndrome and iron overload. *Sci. Rep.* **13**, 16010 (2023). 37749096 10.1038/s41598-023-42550-y PMC10519991

[50] Ghantous, C. M., Azrak, Z., Hanache, S., Abou-Kheir, W. & Zeidan, A. Differential role of leptin and adiponectin in cardiovascular system. *Int. J. Endocrinol.* **2015**, 1–13 (2015). 10.1155/2015/534320 PMC4433709 26064110

[51] Niu, J., Azfer, A., Rogers, L., Wang, X. & Kolattukudy, P. Cardioprotective effects of cerium oxide nanoparticles in a transgenic murine model of cardiomyopathy. *Cardiovasc. Res.* **73**, 549–559 (2007). 17207782 10.1016/j.cardiores.2006.11.031 PMC1855085

[52] Matsuda, M. & Shimomura, I. Increased oxidative stress in obesity: Implications for metabolic syndrome, diabetes, hypertension, dyslipidemia, atherosclerosis, and cancer. *Obesity Res. Clin. Pract.* **7**, e330-41 (2013). 10.1016/j.orcp.2013.05.004 24455761

[53] Pandey, G., Shihabudeen, M. S., David, H. P., Thirumurugan, E. & Thirumurugan, K. Association between hyperleptinemia and oxidative stress in obese diabetic subjects. *J. Diabetes Metab. Disord.* **14**, 24 (2015). 25897417 10.1186/s40200-015-0159-9 PMC4404074

[54] Manna, P. & Jain, S. K. Obesity, oxidative stress, adipose tissue dysfunction, and the associated health risks: Causes and therapeutic strategies. *Metab. Syndr. Relat. Disord.* **13**, 423–444 (2015). 26569333 10.1089/met.2015.0095 PMC4808277

[55] Henning, R. J. Obesity and obesity-induced inflammatory disease contribute to atherosclerosis: A review of the pathophysiology and treatment of obesity. *Am. J. Cardiovasc. Dis.* **11**, 504–529 (2021). 34548951 PMC8449192

[56] Maiolino, G. et al. The role of oxidized low-density lipoproteins in atherosclerosis: The myths and the facts. *Mediators Inflamm.* **2013**, 714653 (2013). 24222937 10.1155/2013/714653 PMC3816061

[57] Navab, M., Reddy, S. T., Van Lenten, B. J. & Fogelman, A. M. Hdl and cardiovascular disease: Atherogenic and atheroprotective mechanisms. *Nat. Rev. Cardiol.* **8**, 222–232 (2011). 21304474 10.1038/nrcardio.2010.222

[58] Ormazabal, V. et al. Association between insulin resistance and the development of cardiovascular disease. *Cardiovasc. Diabetol.* **17**, 122 (2018). 30170598 10.1186/s12933-018-0762-4 PMC6119242

[59] Lopez-Pascual, A., Urrutia-Sarratea, A., Lorente-Cebrián, S., Martinez, J. A. & González-Muniesa, P. Cerium oxide nanoparticles regulate insulin sensitivity and oxidative markers in 3t3-l1 adipocytes and c2c12 myotubes. *Oxid. Med. Cell. Longev.* **2019**, 2695289 (2019). 30863477 10.1155/2019/2695289 PMC6378795

[60] Raman, P. & Khanal, S. Leptin in atherosclerosis: Focus on macrophages, endothelial and smooth muscle cells. *Int. J. Mol. Sci.* **22**, 5446 (2021). 34064112 10.3390/ijms22115446 PMC8196747

[61] Gencer, B. et al. Association between resistin levels and cardiovascular disease events in older adults: The health, aging and body composition study. *Atherosclerosis* **245**, 181–186 (2016). 26724528 10.1016/j.atherosclerosis.2015.12.004 PMC5695677

[62] Park, J. G. et al. Effects of branched-chain amino acids (bcaas) on the progression of advanced liver disease: A korean nationwide, multicenter, retrospective, observational, cohort study. *Med. (U. S.)* **96**, e6580 (2017). 10.1097/MD.0000000000006580 PMC5478300 28614215

[63] Lin, J., Kakkar, V. & Lu, X. Impact of mcp -1 in atherosclerosis. *Curr. Pharm. Des.* **20**, 4580–4588 (2014). 24862889 10.2174/1381612820666140522115801

[64] Matia-García, I. et al. A possible association between the -2518 a>g mcp-1 polymorphism and insulin resistance in school children. *Arch. Endocrinol. Metab.* **62**, 79–86 (2018). 29694633 10.20945/2359-3997000000012 PMC10118690

[65] Chavey, C. et al. Cxc ligand 5 is an adipose-tissue derived factor that links obesity to insulin resistance. *Cell Metab.* **9**, 339–349 (2009). 19356715 10.1016/j.cmet.2009.03.002 PMC2804846

[66] Ellulu, M. S., Patimah, I., Khaza’ai, H., Rahmat, A. & Abed, Y. Obesity and inflammation: The linking mechanism and the complications. *Arch. Med. Sci. AMS* **13**, 851–863 (2017). 28721154 10.5114/aoms.2016.58928 PMC5507106

[67] Asgharzadeh, F. et al. Cerium oxide nanoparticles acts as a novel therapeutic agent for ulcerative colitis through anti-oxidative mechanism. *Life Sci.* **278**, 119500 (2021). 33862111 10.1016/j.lfs.2021.119500

[68] Mohamed, A. A. et al. Inflammatory and endothelial dysfunction indices among Egyptian females with obesity classes i–iii. *Biosci. Rep.* **40**, BSR20192910 (2020). 32893859 10.1042/BSR20192910 PMC7507597

[69] Ponnuswamy, P. et al. Oxidative stress and compartment of gene expression determine proatherosclerotic effects of inducible nitric oxide synthase. *Am. J. Pathol.* **174**, 2400–10 (2009). 19465644 10.2353/ajpath.2009.080730 PMC2684203

[70] Mungrue, I. N. et al. Cardiomyocyte overexpression of inos in mice results in peroxynitrite generation, heart block, and sudden death. *J. Clin. Investig.* **109**, 735–743 (2002). 11901182 10.1172/JCI13265 PMC150906

[71] Fujimoto, M. et al. A role for inos in fasting hyperglycemia and impaired insulin signaling in the liver of obese diabetic mice. *Diabetes* **54**, 1340–8 (2005). 15855318 10.2337/diabetes.54.5.1340

[72] Xia, Y. & Zweier, J. L. Superoxide and peroxynitrite generation from inducible nitric oxide synthase in macrophages. *Proc. Natl. Acad. Sci. U.S.A.* **94**, 6954–8 (1997). 9192673 10.1073/pnas.94.13.6954 PMC21266

[73] Li, J.-M. & Shah, A. M. Endothelial cell superoxide generation: Regulation and relevance for cardiovascular pathophysiology. *Am. J. Physiol. Regul. Integr. Comp. Physiol.* **287**, R1014-30 (2004). 15475499 10.1152/ajpregu.00124.2004

